# NoLogo: a new statistical model highlights the diversity and suggests new classes of Crm1-dependent nuclear export signals

**DOI:** 10.1186/s12859-018-2076-7

**Published:** 2018-02-27

**Authors:** Muluye E. Liku, Elizabeth-Ann Legere, Alan M. Moses

**Affiliations:** 1 Department of Cell & Systems Biology, University of Toronto, Toronto, Canada; 2 Center for Analysis of Genome Evolution and Function, University of Toronto, Toronto, Canada; 3 Department of Computer Science, University of Toronto, Toronto, ON Canada

**Keywords:** Crm1, Export, Nuclear export signals, Algorithms, Variable length motif model

## Abstract

**Background:**

Crm1-dependent Nuclear Export Signals (NESs) are clusters of alternating hydrophobic and non-hydrophobic amino acid residues between 10 to 15 amino acids in length. NESs were largely thought to follow simple consensus patterns, based on which they were categorized into 6–10 classes. However, newly discovered NESs often deviate from the established consensus patterns. Thus, identifying NESs within protein sequences remains a bioinformatics challenge.

**Results:**

We describe a probabilistic representation of NESs using a new generative model we call NoLogo that can account for a large diversity of NESs. Using this model to predict NESs, we demonstrate improved performance over PSSM and GLAM2 models, but do not achieve the performance of the state-of-the-art NES predictor LocNES. Our findings illustrate that over 30% of NESs are best described by novel NES classes rather than the 6–10 classes proposed by current/existing models. Finally, many NESs have additional hydrophobic residues either upstream or downstream of the canonical four residues, suggesting possible functionality.

**Conclusion:**

Applying the NoLogo model highlights the observation that NESs are more diverse than previously appreciated. Our work questions the practice of assigning each NES to one of several predefined NES classes. Finally, our analysis suggests a novel and testable biophysical perspective on interaction between Crm1 receptor and Crm1-dependent NESs.

**Electronic supplementary material:**

The online version of this article (10.1186/s12859-018-2076-7) contains supplementary material, which is available to authorized users.

## Background

Modeling short patterns of DNA or Protein residues is a major research area in bioinformatics. These short patterns, or motifs, are important for many types of biological interactions, particularly in regulatory networks. The main model currently used for short motifs is the so-called position specific scoring matrix(PSSM) or position weight matrix (PWM) model [1], typically represented as a sequence logo [2, 3]. These models assume that motifs are composed of a fixed number of independent positions, and estimate parameters to describe the residue preferences in each position. Despite their wide use and many advantages, there are many biological sequence families for which this motif model is a poor description. Here, we propose a new generative model for an important example of a short protein motif that does not fit well to the standard models: the Nuclear Export Signal (NES).

Crm1-dependent Nuclear Export Signals (NESs) are short, weak, variable length patterns found in proteins that are critical for recognition of cargo (or target) proteins for nuclear export by the receptor Crm1. The first studies characterizing NES signals identified peptide sequences both necessary and sufficient for export in PKIalpha and HIV Rev. [4, 5]. The salient feature of these NESs is the clustering of Leucines and other hydrophobic amino acids within a window of 10–15 amino acids as originally observed [4, 6]. Subsequently it was determined that Crm1 was the transport protein for these signals [7–11]. Later studies on Crm1 dependent NESs expanded the possible patterns of amino acids that can function as an NES and suggested the substitution with more amino acids [12, 13]. Structural studies of the Crm1 bound to NES peptides, revealed unexpectedly that in addition to four hydrophobic amino acids of the NES peptide that there was a fifth hydrophobic pocket in Crm1 that bound a fifth hydrophobic amino acid [14, 15]. Additionally, another structural study showed that some peptides can bind in a reverse orientation (C-term to N-term) [16]. The apparent large variability in NES sequences and apparent flexibility of Crm1 and NES peptide binding, suggest that traditional lock and key mode of substrate-receptor interaction fails to explain the observed data.

Previous attempts to model the sequence diversity of the Crm1 dependent NESs computationally have employed consensus sequences in the form of regular expressions, PSSMs or both [6, 13, 17–19] as one aspect of their feature space. Since the weak patterns and variable lengths of NESs are not all captured by various regular expressions proposed nor by a single PSSM model (which is based on an underlying assumption of a single lock and key type of interaction between the Crm1 receptor and its substrates) previous bioinformatics studies employed up to six PSSMs or regular expressions to represent the sequence diversity of Crm1 dependent NESs. To reduce the high false positive rate, they have had to incorporate other meta-features of NESs such as disorder propensity, secondary structure information and evolutionary conservation among others into machine learning algorithms like support vector machines (SVM) or neural nets. Yet the false positive rates remain high, such that predicting NESs on a proteome-wide scale becomes prohibitive when considering experimental verification [6, 18].

We present a new generative probabilistic model that can account for the sequence variation of Crm1 dependent NESs. The key aspect of our model, relative to the above-mentioned models, is that it allows the NES to assume multiple binding configurations and is not limited to the six or so canonical NES classes. Our model is most similar to Wregex [19], which combines regular expressions and PSSMs to give quantitative scores to matching sequences. Our model (which we call NoLogo) can be viewed as a probabilistic generalization of Wregex, and can therefore be trained using standard algorithms. We show that NoLogo uses fewer parameters, but is comparable in its predictive power to the best (to our knowledge) probabilistic variable length motif model (GLAM2) and exhibits higher recall rate compared to NES specific predictors that rely mostly on primary sequence information. Furthermore, taking advantage of the interpretability of our model, we demonstrate that the six to ten categories of NESs currently used cannot account for all the variety of experimentally verified NESs. Finally, we show statistical evidence that a substantial number of known NESs have an additional hydrophobic amino acid as suggested by the small number of crystal structures [14–16].

## Results

### A collection of well-characterized *S. cerevisiae* NESs

In order to train our algorithm, we sought to obtain a set of Crm1-dependent Nuclear Export Signals (NESs) where we could be as confident as possible about the biochemical and genetic evidence supporting the precise sequence of the NES. Although there are well-established collections of Crm1-dependent Nuclear Export Signals [12, 20–22], they differ in the inclusion criteria, experimental techniques, cell-lines, and delineation of motif boundaries. Previous bioinformatics approaches to model NESs have used various approaches to define the exact coordinates of the NES instances in protein sequences in the training sets.

We therefore decided to curate a collection of known NESs from the budding yeast literature. This approach has several advantages. First, since these NESs are all from a single species, there is no issue with inclusion of redundant orthologous NESs. Second, these NESs are from a unicellular organism, so we can be confident that NESs are identified in a relevant in vivo cellular context. Finally, and perhaps most importantly, convenient genetic molecular biology tools in *S. cerevisiae* mean that the experimental evidence is relatively reproducible and complete.

**Fig. 1:**
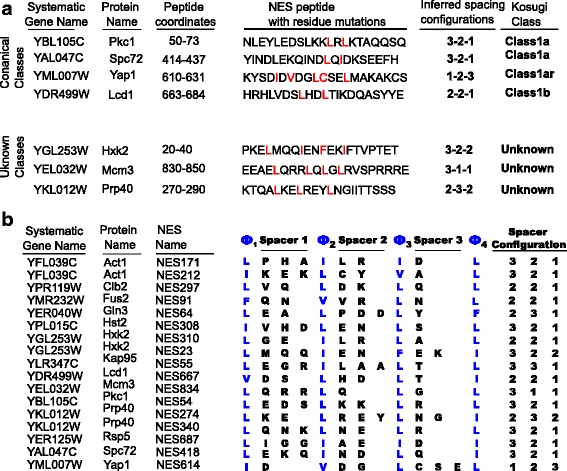
Analysis of high-confidence *S. cerevisiae* NESs. **a** shows unaligned 21 amino acid peptide window of 4 yeast NESs with canonical spacing configurations (upper panel) and 3 yeast NESs with spacing patterns that are inconsistent with any of the canonical spacing configurations (lower panel). The residues demonstrated through mutagenesis studies to be important for NES function are highlighted in red. (See Additional file 1: Table S1 for details) **b** NESs manually aligned with gaps based on their hydrophobic positions. The numbers in the NES names refer to amino acid position of first hydrophobic residue

We obtained two sets of NESs, which we call 17hcScNES (17 high confidence *S. cerevisiae* NESs) and 35ScNES (35 *S. cerevisiae* NESs) defined as follows. The 17hcScNES are in 14 proteins that meet the following criteria. The NES candidate sequence had to (i) be narrowed down to within a short stretch of 25 or less amino acids, and the studies had to demonstrate (ii) necessity, (iii) sufficiency and (iv) Crm1 dependence. We then defined a less stringent set, 35ScNES, which contained 17hcScNES, as well as 18 other NESs from *S. cerevisiae* where two of the four criteria were met. NESs are poorly described by sequence logos [2, 3] and therefore are not amenable to motif sequence logo analysis. NESs have been categorized to as many as ten classes [18, 23] based on their sequence patterns of hydrophobic residues. Many well-characterized NESs from our collection of *S. cerevisiae* NESs fall into these classes (Fig. 1a, top panel). However, several NESs do not fall into any of the previously described classes (Fig. 1a bottom panel). In these cases, site-specific mutagenesis data on the hydrophobic residues necessary for NES activity rules out the previously defined classes (references to primary literature supporting these NESs and more details are provided in Additional file 1: Table S1).

In contrast, our hand alignment of the 17hcScNES (Fig. 1b) and 35ScNES (Additional file 2: Figure S1) show, as expected, that the NESs contain ~ 4 hydrophobic residues as well as a slight preference for negatively charged “spacer” residues [12, 13, 17] between the hydrophobic positions. As expected, these well-characterized NESs show variation in length from 9 to 15 amino acids, the pattern contains few high-information content positions unless a gapped alignment is made (Fig. 1b). We note that the length variability in NESs arises from the spacer regions: the hydrophobic positions are usually single positions, while the spacers vary from 1 amino acid to 3 amino acids in length. This is consistent with experimental information from site-specific mutagenesis studies [4, 6, 12, 19, 24].

### NoLogo: A generative, variable length model for NESs

Because NESs show variability in length, they are not well-modelled by the position-specific scoring matrix (PSSM), the standard generative probabilistic model for biological motifs [25, 26]. Current bioinformatics approaches use a collection of multiple PSSMs and regular expressions to capture the diversity of NESs, but it is recognized that there are known NESs that fall outside of these known patterns [6, 17–19]. To highlight this problem Fig. 1a illustrates three NESs whose site-specific mutagenesis studies suggest novel spacer configuration patterns and those of canonical spacer configuration patterns. We therefore sought to develop a new probabilistic model for NESs based on the insight that the variability in NES length is due to the variability in the spacer lengths. If we knew the lengths of the three spacers in a given NES (which we refer to as the spacer configuration, *C*), we could write the probability of an NES as follows:

1 $$ P\left(X|C\right)=\prod \limits_{i\in {\Phi}_C}{p}_{\Phi}\left({X}_i\right)\prod \limits_{j\in {S}_C}{p}_S\left({X}_j\right) $$

Where *X* represents an amino acid sequence, *i* and *j* index the hydrophobic and spacer positions, respectively, for the configuration, *C*, and we have introduced categorical probability models for the residues in the hydrophobic positions (*p*_Φ_) and the spacer positions (*p*_S_). Although we can attempt to define a single “true” configuration for an NES using a hand-alignment or regular expression matching, the lengths of the spacers are not known for most NESs. We therefore define the NoLogo model by treating the spacer configuration as a hidden variable, and marginalize over it:

2 $$ P\left(X| NoLogo\right)=\sum \limits_CP\left(X|C\right)P(C) $$

**Fig. 2:**
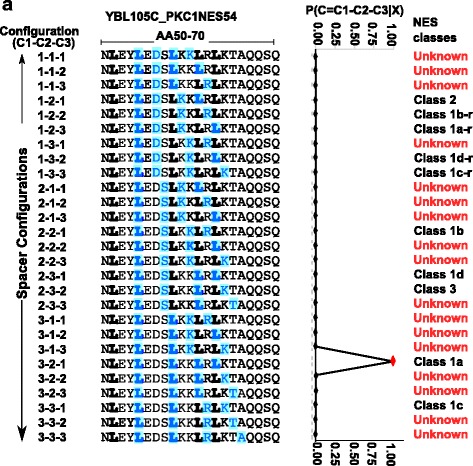
Twenty seven possible configurations for the Pkc1 NES. Each row corresponds to a possible spacer configuration. C1-C2-C3 indicate the lengths of the three spacers. Bold letters in the amino acid sequence correspond to hydrophobic residues. Letters in blue correspond to the letters in the hydrophobic positions in the corresponding configuration. P(C = C1-C2-C3 |X) is the posterior probability of the configuration under the NoLogo model. Posterior probabilities for configurations are indicated by the line graph adjacent to the sequences. Currently accepted NES classes along with unknown classes are indicated in the right column

Where the sum is over the set of all allowed spacer configurations, weighted by their probabilities. For this work, we decided to allow lengths of 1, 2 or 3 amino acids at each of the three spacer positions, yielding 27 possible configurations (Fig. 2 illustrates the idea of summing over spacer configurations). Thus, the NoLogo model is a 27-component mixture model, but each component of the mixture shares the parameters for the amino acid probabilities at the hydrophobic and spacer positions. We assume that each of the spacer lengths are chosen independently, so that we can write the probability of the configuration, *C*, as the product of the probabilities of the lengths of each of the three spacers, *C*_*1*_, *C*_*2*_, *C*_*3*_:

3 $$ P(C)=P\left({C}_1|{p}_{C1}\right)\times P\left({C}_2|{p}_{C2}\right)\times P\left({C}_3|{p}_{C3}\right) $$

Where we introduce a categorical probability model for the spacers, *p*_*C*_, which is a 3 × 3 matrix of parameters, such that *p*_*Ca*_ is a vector of probabilities at spacer position *a*, and *p*_*Cab*_ is the probability that the a-th spacer has length *b*. In practice, to ensure that the probabilities of the configurations are comparable, we also include residues generated by a background distribution so that we are always computing the probability of 13 amino acids total. In the case of PKC1, as illustrated in Fig. 2, the NES is best described by the “3–2-1” configuration as suggested by the greater than 0.99 posterior probability in the “3–2-1” spacing configuration.

### Parameter estimation for the NoLogo NES model

**Fig. 3:**
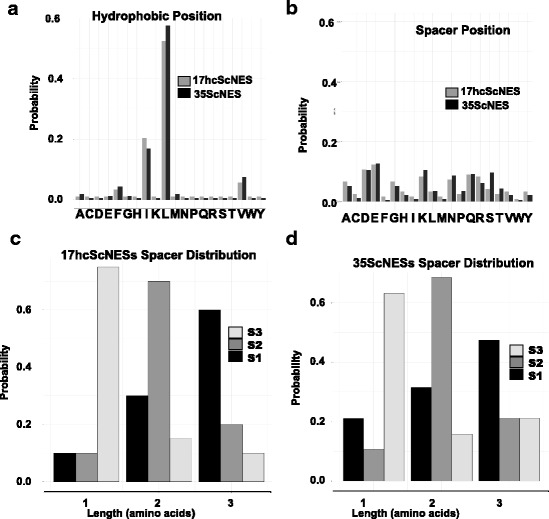
NoLogo model parameters for amino acid residues (**a**, **b**) and spacer lengths (**c**, **d**) estimated from manual hand alignment (**a**-**d**). S1, S2 and S3 respectively indicate Spacer 1, Spacer 2 and Spacer 3 in Fig. 1c-d. See text for details

The model defined above includes two vectors of amino acid probabilities (20 parameters each, 19 of which are free parameters) as well as 3 vectors of spacer probabilities (3 parameters each, 2 of which are free parameters) for a total of 44 free parameters. We note that a PSSM model of length 10 amino acids has 10 vectors of amino acid probabilities, for 190 free parameters. Thus, although our model is slightly more complicated in terms of computation, it should, in principle, require less data to train. We first trained the NoLogo model using the hand aligned sets of *S. cerevisiae* NESs described above. We assumed that the hand alignment specified the “true” configuration of each NES and from this we derive the parameters for each amino acid at the hydrophobic position and spacer position and spacer length probabilities. We also include a pseudocount of 1 per position to smooth the amino acid and spacer probability models. Figure 3a-b shows a comparison of hydrophobic and spacer position parameters inferred from the hand alignments of 17hcScNES and 35ScNES. As expected, the hydrophobic positions are dominated by leucine residues, while the spacer positions show a weak preference for acidic amino acids. In addition, the spacer probabilities derived from 35ScNESs and 17hcScNES show that the most probable spacer configuration is “3–2-1”, meaning that the first spacer is of length 3, the second spacer is of length 2 and the third spacer is of length 1 (Fig. 3c and d). This is consistent with both classical work [13]and more recent analysis of larger numbers of NESs, where “3–2-1” configuration is the most prevalent [6, 17–19].

Although consistent with previous knowledge of NESs, the NoLogo models inferred from the hand alignments are potentially biased by the expert aligner. Because the NoLogo model is a generative probabilistic model, it is possible to estimate parameters from un-aligned sequences using expectation maximization (E-M) (see Additional file 3), similar to what has been used for motif-finding [27]. If we start with uniform initial estimates of the spacer parameters, this approach is then unbiased with respect to our expectations about the configurations of NESs. We implemented an E-M algorithm, and found similar estimates of the parameters to those obtained from the hand alignment, although the E-M algorithm shows relatively higher frequency of 3–2-1 spacing configuration (Additional file 4: Figure S2). Nonetheless, this indicates that the accepted “consensus” for NESs can be obtained from the data in an unbiased way.

### Comparison of predictive power of the NoLogo NES model to a PSSM, an HMM-based model, Wregex, NESmapper and LocNES

Despite more than a decade of progress, predictions of NESs from primary amino acid sequences remains a current research challenge [23]. We therefore sought to test whether the NoLogo NES model that we proposed could offer improvements in NES prediction. Because the NoLogo model is a generative probabilistic model, to use it for prediction we simply compute a log-likelihood ratio score, llr:

4 $$ llr=\log \frac{P\left(X| NoLogo\right)}{P\left(X| bg\right)} $$

**Fig. 4:**
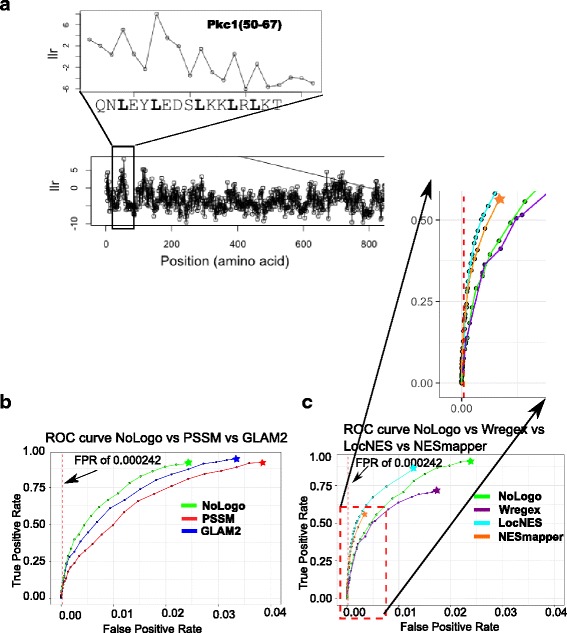
Predicting NESs using the NoLogo model. **a** shows the log likelihood ratio score over a portion of Pkc1, inset is the region corresponding to the known NES. **b** shows ROC curves of NoLogo prediction performance on the NESdb vs PSSM and GLAM2 respectively. The dotted red line indicates the false positive rate, at which we evaluate the predictions at a proteome wide level. **c** The relative prediction performance of NoLogo, Wregex, LocNES and NESmapper, by ROC curve analysis. Above is a zoomed-in image of the bottom left quadrant of the graph representing the true positive rates at low false positive rates. See text for details

Where *P*(*X*|*NoLogo*) is, the probability defined above (formula 2), and *P*(*X*|*bg*) is the probability of the sequence given a background model where the probabilities of the residues are estimated based on the frequencies in the entire yeast proteome (see Methods). This statistic is directly analogous to that used for prediction of motif instances using PSSM models [28]. An example of this likelihood ratio score for a protein containing a known NES is shown in Fig. 4a. The rank of PSSM score feature was the single best discriminative feature in a state-of-the-art NES predictor [6]. So, we sought to determine the relative performance of the NoLogo model to a PSSM model. We estimated a PSSM model from the hand alignment of *S. cerevisiae* NESs (see Methods) and compared the prediction performance (see Methods) on the NESdb, a large set of NESs from multiple species that was not used in training the models [22]. We found that the NoLogo model predicted significantly more true positives (at false positive rates greater than 0.005 with P.value < 0.05, see Methods) compared to the PSSM model (Fig. 4b) indicating that it is capable of capturing more of the true diversity of NESs. We also assayed the effect of training set size and confidence by using either the 17hcScNES or the 35ScNES to train NoLogo NES models and found that the overall performance was similar. We also performed a more systematic analysis of training set size and prediction performance ROC curve (Additional file 5: Figure S3a, see Discussion).

A classical bioinformatics approach to model protein motifs of varying lengths is using HMMs [29]. We therefore next compared the NoLogo model to GLAM2, an HMM-based approach that can identify weak motifs in unaligned protein sequences [30]. GLAM2 trains general HMMs that have amino acid probability parameters at each position of the motif by relying on prior distributions and has been shown to generate highly specific models for short linear motifs [30]. Although there was considerable variability in the motif models obtained from GLAM2 (Additional file 6: Figure S4) based on the 35ScNES, we were able to obtain models that showed predictive power that was better than PSSM but showed less predictive power than NoLogo on the NESdb within a false positive rate range from 0.00042 to 0.03 with p.value< 0.05 by Fisher’s exact test (Fig. 4b). This indicates that the NoLogo model offers similar if not better predictive power to HMMs, albeit with fewer parameters (see Discussion). The state-of-the-art NES prediction method, locNES [6], shows much greater predictive power on the NESdb than NoLogo NES model that we trained (Fig. 4c). The range of false positive rate in which LocNES appears to perform better and is statistically significant is 0.0003 to 0.01 with P.value < 0.01. Because locNES was trained on the NESdb, we sought to test whether this performance advantage was due to circularity in the analysis. We compared locNES and NoLogo on a set of 19 yeast NESs that were not available for training for either method. Although the sample size is too small to reach statistical significance, we found similar trends in performance of the methods (Additional file 7: Figure S5) suggesting that the results we obtained on NESdb are not the results of locNES overfitting to the training set. We extended the comparison on NESdb to Wregex and NESmapper (Fig. 4c) and found the following results. NoLogo outperforms Wregex at false positive rates of greater than 0.005 with *P*-values less than 0.008, which included the relaxed setting of Wregex to increase the number of predictions made. NESmapper outperforms NoLogo at false positive rates of 0.006 to 0.004 at P-values less than 0.01, but its recall rate plateaus at 56%. We note that NESmapper does not rely on only primary sequence alone but incorporates NES flanking regions properties to predict NESs. This could partially explain the improved performance of NESmapper over NoLogo at lower false positive rate. Once again, analysis on a collection of yeast NESs that was not available to any of the methods did not produce statistically significant differences in performance (Additional file 7: Figure S5), We initially evaluated all the algorithms at a false positive rate (FPR) of 0.000242, which would be needed to achieve proteome-wide predictive power (see Methods, indicated as the dotted vertical line on Fig. 4b, c, Additional file 5: Figure S3 and Additional file 7: Figure S5). However, we found that at that level of specificity, there were no statistically significant differences between the algorithms, and the NES recall rate is very low. Thus, we decided to report the range of false positive rates within which, there are statistically significant differences in performance. This is also evident graphically in the ROC curves. The inability to determine a difference in performance at a false positive rate of 0.000242 suggests that achieving proteome-wide predictive power for NESs remains a challenge for current bioinformatics methods.

### NoLogo NES model suggests new classes and multiple configurations of NESs

Because the NoLogo NES model allows NESs to occupy all 27 allowed spacer configurations, we next sought to use the NoLogo model to discover which spacer configurations were actually used by known NESs, and to test whether NESs typically use a single configuration at all (based on the patterns of hydrophobic amino acids.) To do so, we identified the most probable starting position for each known NES (using the likelihood ratio score, see Methods) and computed the posterior probability for each of the 27 configurations at that position as follows:

5 $$ P\left(C|X\right)=\frac{P\left(X|C\right)P(C)}{P\left(X| NoLogo\right)} $$

**Fig. 5:**
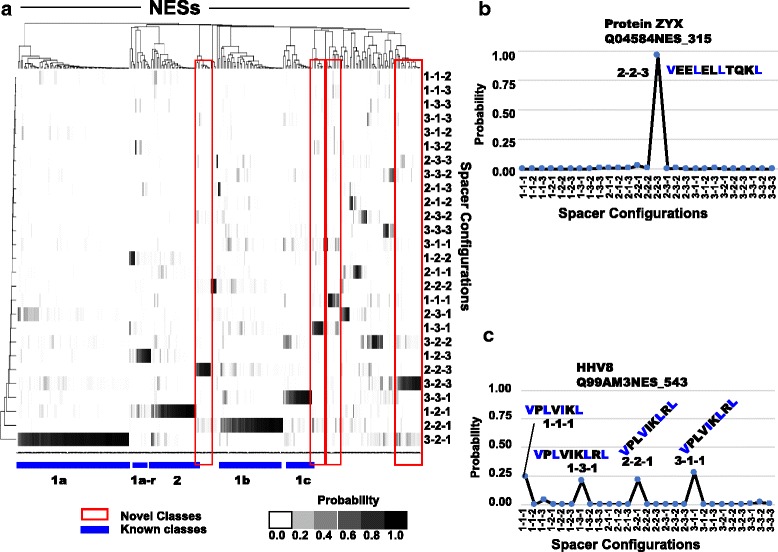
Clustering of NESdb identifies known and novel classes. **a** Posterior probabilities of NES configurations are indicated in a heat map, where black corresponds to higher probabilities. Two hundred seventy-nine NESs have been clustered based on similarity, while configurations are ordered from most populated to least (bottom to top). Clusters corresponding to previously known classes of NESs are indicated as blue lines, while novel classes are indicated as red boxes. **b** shows an example of a high scoring NES that clearly matches unknown or undescribed class of spacing configuration 2–2-3. **c** shows an example of an NES that shares equal probability (approximately 0.2) in 4 different classes of spacing configurations; 3 Novel (1–1-1, 1–3-1, and 3–1-1) and 1 of the canonical classes (2–2-1)

To explore the “spacer configuration space” we clustered these posterior probability vectors for the NESdb and displayed the results as a heat map (Fig. 5). Impressively, we found that the 5 of the 6 so-called Kosugi classes of NESs [12] and the reverse of class 1a–d [16] represent 6 of the 11 most common classes of NESs in the NESdb (Fig. 5a). Nevertheless, these classes do not capture the true diversity of the NESdb: we found 31.3% of NESs fall within 5 additional new classes (Fig. 5a). For example, an NES in ZYX (Uniprot ID: Q04584) shows > 0.9 posterior probability for a 2–2-3 configuration (Fig. 5b). We also found that 18% of NESs have > 0.2 posterior probability in more than 1 configuration. These included an NES in HHV8 (Uniprot ID: Q99AM3) which exhibited ~ 0.2 posterior probability in 4 almost exclusively novel configurations (Fig. 5c). This suggests that these NESs may interact with Crm1 through a dynamic interaction, rather than the classical lock-and-key model for receptor substrate interaction (see Discussion). To confirm that the observed clustering was not the result of bias introduced by our hand alignment, we repeated this analysis using the parameters obtained through E-M and found similar results (Additional file 8: Figure S6), although there were more cases of NESs falling in novel classes relative to hand alignment. To test whether the novel configurations were needed to describe NESs, we restricted the space of spacer configurations to the six Kosugi classes [18] and the four additional reverse classes [23]. We find that most of the NESs in NESdb could be described by these 10 classes (Additional file 9: Figure S7). With this model we found that 14.7% of NESs now have > 0.2 posterior probability in more than 1 configuration (compared with 18% for the unrestricted NoLogo model, Additional file 10: Figure S8a). Some NESs that were described by multiple novel configurations (Fig. 5c), collapse into a single configuration when we restrict to 10 canonical classes (Additional file 10: Figure S8c). Nevertheless, 14.7% of NESs are still best described by multiple class membership. An example of an NES that is classified as having greater than 0.2 posterior probability in more than 1 configuration is that of the alpha-2 catalytic subunit of AMP-activated protein kinase (Additional file 10: Figure S8d), which also had multiple class membership when the NoLogo model allowed 27 possible spacer configurations. Some of the NESs that were previously described by a single novel spacer configuration (Fig. 5b) are now classified into one of the canonical classes (Additional file 10: Figure S8b), often due to a change in the start position of the NES. Interestingly, even the NESs non-canonical spacer lengths in Fig. 1a can be assigned to canonical classes if other start positions and residues other than the experimentally defined hydrophobic positions are included. These results suggest that the possibility of alternative start positions and hydrophobic positions complicate the definition and prediction of NESs (see Discussion).

### NoLogo NES model reveals additional hydrophobic positions in known NESs

The ambiguity in defining the hydrophobic residues is consistent with structural studies of NESs bound to Crm1 [14–16] revealed that, unexpectedly, Crm1 accommodates a fifth hydrophobic amino acid in addition to the standard four for NESs like SNUP1. Consistent with this, using multiple nearby predictions of NESs improves NES prediction accuracy overall [6]. Furthermore, during the calculation of log-likelihood ratio score for NESs in protein sequences using NoLogo we observed instances in which a hydrophobic residue, typically 3 to 4 amino acids upstream of the true NES start site, exhibited a high likelihood ratio score of being an NES. Moreover, in our analysis of predictive power, we noted that the best performing GLAM2 models (albeit not the ones with the highest score, see Discussion) included 5 hydrophobic positions, rather than the standard four. We therefore sought to use our new model to test whether known NESs tend to contain additional hydrophobic residues. Because the NoLogo does not assign an NES to a single spacer configuration, we can use the model to estimate the probability that each hydrophobic residue is used as part of an individual NES. To do so, we computed the posterior probability that a residue, at position *k*, is a hydrophobic position as follows.

6 $$ P\left({X}_k\  is\ hydrophobic| NoLogo\right)=\sum \limits_{i=1}^WP\left( NES\  starts\  at\ i|X\right)\sum \limits_CP\left(C|X\right)H\left(C,k-i\right) $$

Where *C* indexes the spacer configurations, *H*(*A*, *b*) is an indicator variable that takes 1 if an NES in configuration *A* has a hydrophobic position at *b*, or 0 otherwise. *P*(*NES starts at i*|*X*) is the posterior probability that the NES begins at position *i* and is computed as follows.

7 $$ P\left( NES\  starts\  at\ i|X\right)=\frac{P\left({X}_i| NES\  starts\  at\ i, NoLogo\right)}{\sum_{j=1}^WP\left({X}_j| NES\  starts\  at\ j, NoLogo\right)} $$

Where *W* are the possible starting positions within a peptide window containing the experimentally defined NES. For the experiments described here, we used 25 residue windows where the NES can start within the first 12 residue positions.

**Fig. 6:**
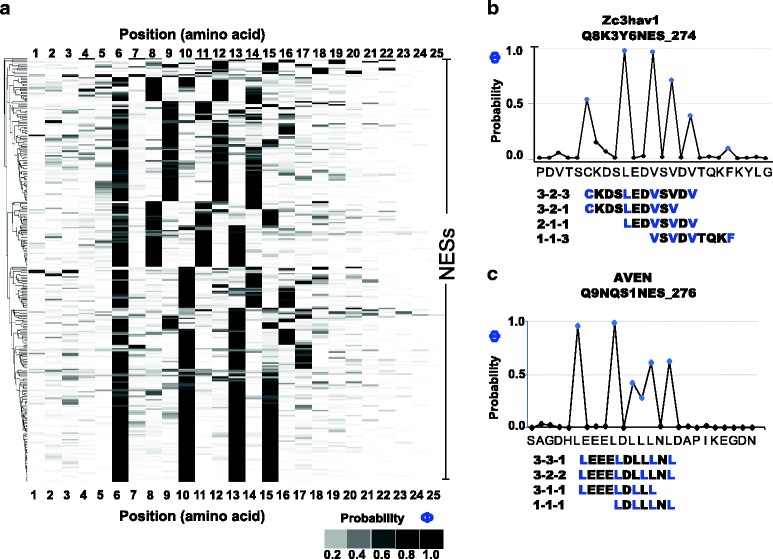
Clustered heat maps of the posterior probabilities of being a hydrophobic position within each of the subset of NESdb NESs analyzed as the 25amino acid peptide window. Panel (**a**) The columns represent the amino acid position and the rows of the heat map represent the individual NESs. The legend shows the grey scale intensity with corresponding probability for the heat map. Panel (**b**) and (**c**) Shows two example NESs with plots of the posterior probability of being a hydrophobic position for each of the residues in the 25-amino acid window. Also included are the possible configurations that can be assumed using different combinations of the most probable hydrophobic residues

We computed the posterior probability of being a hydrophobic residue for each residue of the NESs in the NESdb where the experimentally defined NES window was less than 25 amino acids (266 NESs). Although 25 amino acids length is an arbitrary threshold, it is in the amino acid range context we suspect to harbor NES activity. We then clustered these posterior probability vectors and displayed the results as a heat map. We found that out of 266 NESs 101(which have at least 0.2 probability in all 4 positions) have the 3–2-1 spacer configuration. Of these, 71.3% (72 NESs) show 4 strong hydrophobic positions (which have greater than 0.4 probability), consistent with the classical NES consensus sequence. Figure 6 shows the heat map representation of the posterior probability profiles for 266 NESs from NESdb aligned so that the best-scoring position (according to the llr) is defined to be 6 (with the exception of N-terminal and C-terminal NESs). The analysis of posterior probability of being a hydrophobic residue, reveals that not only do NESs occupy multiple spacer configurations, there are often additional hydrophobic residues immediately upstream or downstream of the best scoring NES. Overall, we found 34.6% of NESs with at least 0.2 posterior probability in 5 residues. The same analysis for the 35ScNESs yielded 31.4%, which is in the same range. Inspection of individual examples (Fig. 6b, c) confirms that there is substantial ambiguity in the assignment of hydrophobic residues, even for NESs that can be assigned to the previously defined consensus classes. This suggests that there is variability either in the number of residues that contact the Crm1 receptor, or that some NESs may adopt multiple binding states or interfaces that correspond to different spacer configurations and starting positions (see Discussion).

## Discussion

NESs are difficult to define because performing empirical mutational studies of different permutations of hydrophobic and spacer patterns is prohibitive, especially since most of the studies’ goal is to show that a protein is an export substrate and not necessarily to understand the mechanism of how the substrate interacts with Crm1. Consequently, most of the experimental studies employ deletion of the whole region encompassing candidate NES or mutate all the suspected hydrophobic residue positions. Therefore, to train our model, we curated a small set of high-confidence NESs from the *S. cerevisiae* literature. We did not bias our criteria to NESs that fit the standard patterns [12], instead we let ourselves be guided by the experimental evidence establishing Crm1 dependent NES activity. Although these 17-high confidence NESs confirmed expected patterns of residues, we found they were not critical for training the NoLogo NES model: we obtained similar models, regardless of the training data used.

We have presented a new probabilistic model (NoLogo) for predicting Crm1-dependent NESs that has fewer parameters, but performs better than other generative models (GLAM2s HMM-model and a PSSM model). Our model is designed to capture the repeating residue pattern within the weak motif. We estimate parameters for residue classes rather than positions, and model the spatial relationship between the residues classes using a simple model of the spacing between classes. The model uses less parameters because it shares information between residue classes, which is in contrast to the approach taken in GLAM2, where a parameter-rich, position specific model is estimated from small datasets by relying on prior distributions. Consistent with our assertion that the NoLogo model requires little training data, we showed that it demonstrates acceptable predictive power when even as few as 5 known example NESs were used to train it (Additional file 5: Figure S3a). This performance with 5 known NESs was comparable to that of our PSSM model (Additional file 5: Figure S3a). We believe that this model could be applied to other weak, but important, biological motifs that do not have a clear position specificity, such as nuclear localization signals (NLSs). Compared to other NES specific predictors (NESmapper, Wregex, and LocNES) the NoLogo NES shows overall less predictive power at low false positive rates than the state-of-the-art methods. However, we find that NoLogo has higher recall rates (Fig. 4c). Unlike state-of-the-art machine learning models like neural networks and SVMs, an advantage of our model is that it is an interpretable generative probabilistic model, whose internal states (configurations) and parameters (residue frequencies) correspond to biological properties of the NESs. On the other hand, our model does not consider the variety of Crm1 dependent NES features measured by the various groups, such as solvent accessibility context of NESs, disorder propensity, hydrophobicity index, etc. We believe that the predictive power of our model could be improved by including these other features. In this study, however, we exploited the interpretability of the probabilistic model to test several hypotheses about known NESs.

One hypothesis is to ask, using the NoLogo NES model, how many configurations were represented by the data. We found that the data is consistent with more than the previously accepted 6–10 classes of NESs [18, 23]. This corresponds to a biophysical picture where multiple binding configurations are possible for the NES-Crm1 interaction, which is consistent with the surprising diversity found in the few known structures solved for NESs [14–16, 23, 31], and suggests that there are likely to be many more possible binding configurations. This is consistent with the idea that the Crm1-cargo interaction is much more flexible than traditional lock-and-key models of receptor-substrate interaction. When we constrain the model to the previously accepted classes, we find similar predictive power (Additional file 9: Figure S7), but NESs are forced to adopt non-canonical hydrophobic residues or alternative start positions in order to fit the previously accepted classes. In general, we found additional hydrophobic positions with high probability immediately adjacent to known NESs. This means that defining the exact boundaries of these motifs will continue to be a challenge for bioinformatics models that require training data.

Finally, we found individual NESs that showed high probability for multiple configurations, consistent with the hypothesis that some NESs might interact dynamically, switching between conformations in “fuzzy” complexes [32, 33]. Occupancy in multiple configurations was observed even when we restrict the possible spacer configurations to just 10 canonical classes.

Since dynamic interactions have been proposed for disordered peptide binding, and NES prediction algorithms proposed that disorder context helps distinguish true NESs from false predictions [6, 17], we tested whether our known NESs were in disordered regions. Counter to what we expected, we found that many of the NESs in the training set did not fall in a clearly defined disordered region(according to DISOPRED3 prediction) [34]. During preparation of this manuscript, structural work [23] demonstrated a surprisingly diverse and dynamic structural conformations adopted by NESs when bound to Crm1, with the only common feature being a single helical turn that binds the central region of the binding pocket. This work lends strong support and credibility to our proposition that some NESs may adopt multiple configurations and therefore interact with Crm1 in a dynamic fashion where each encounter may lead to a different interaction interface.

Our observations are also consistent with the suggestion that Crm1 dependent NESs are generally weak interacting motifs [35]. One ramification for weak binding threshold is expansion of what patterns are permitted, consistent with the wide diversity of Crm1 dependent NES. This is in contrast to strong motifs in which the sequence pattern is invariant and strict and one structural study can be representative of the whole group.

## Conclusion

In conclusion we have provided a novel generative probabilistic model for predicting Crm1-dependent NESs. We demonstrate that it performs quantitatively better than PSSMs and general HMM models, has higher recall rate than the NES specific predictors benchmarked in this study, but this comes at a cost of reduced predictive power. We believe that incorporation of NoLogo predictions as a feature into machine learning algorithms would lead to more accurate prediction of Crm1-dependent NESs. Lastly, our analysis suggests that many NESs do not fit the canonical classes (or must be forced to adopt an alternative start site to fit the canonical class), have membership in multiple classes and tend to have additional hydrophobic residues nearby.

## Methods

### Selection of training set and test set NESs

*S. cerevisiae* NESs used as training set in this study along with the literature references that provide evidence in support of NES function and summaries of findings are found in Additional file 1: Table S1. The NESdb NESs, which were used as the test set for algorithm performance evaluation, and their amino acid coordinates in their respective proteins were exactly the same ones provided in the supplementary table of Xu D. et al. [6] but we removed ten of the NESs from this set since this subset was used in the *S. cerevisiae* training set NESs (35ScNESs). Thus, the total NESs in the NESdb test set were 279. In the effort to compare NoLogo with LocNES, NESmapper, and Wregex on a set of NESs that do not overlap with the training set of these algorithms (Additional file 7: Figure S5), we partitioned the 35ScNESs into a 15ScNESs as training set and 19ScNESs as test set. The 15ScNES training set contain the 10 yeast NESs that were found in the NESdb, which was used to train LocNES, and random sampling of 5 additional ScNESs to stabilize the performance of NoLogo (YPL015C, YDR499W, YEL032W, YER125W, YAL047C, YML007W, YKL143W, YKR048C, YHR170W, YMR235C, YMR232W, YLR131C, YOL111C, YIR006C, YOR329C; See Additional file 1: Table S1 or Additional file 2: Figure S1). The remaining NESs were included in the 19ScNESs test set (These consisted of YBL105C, YDL207W, YDL229W, YER040W, YER165W, YFL039C, YGL071W, YGL253W, YJL128C, YJL187C, YKL012W, YLR074C, YLR347C, YPR093C, YPR119W; See Additional file 1: Table S1 or Additional file 1: Figure S1).

### Estimation of PSSM models for *S. cerevisiae* NESs

For the PSSM model we performed an eleven-amino acid alignment and a twelve-amino acid alignment encompassing each of the identified NESs. This was the minimal sequence length that allowed us to capture all the critical residues for export determined by experimentation for these 35ScNESs. After aligning all NESs in a twelve-amino acid window, we counted the frequency of each amino acid’s appearance in that position divided by total number of NESs. This gave the probability parameter of each amino acid at each position. We smoothed these estimates by adding a pseudocount of 1 per amino acid at each position.

To identify matches to the PSSM, we use the standard likelihood ratio score [28] to compare the probability of the sequence under the PSSM model to a non-position specific background model. The probability parameters for the background model were derived by counting the relative frequency of each amino acid in the entire *Saccharomyces cerevisiae* proteome. These background probabilities are also the same ones used in the NoLogo background model. These values are provided in Additional file 11: Table S2.

### Estimation of NoLogo models for *S. cerevisiae* NESs

The NoLogo model parameters are derived from all 35 *Saccharomyces cerevisiae* NESs (see Additional file 2: Figure S1) and in some cases from a subset of these 35ScNESs (see Fig. 1b). Applying the NoLogo model to interrogate protein sequences requires two sets of parameters. The first is the set of amino acid frequencies in the hydrophobic position. After manually aligning all NESs by choosing a starting hydrophobic position and a spacer configuration, we counted the frequency of each amino acid’s appearance in the hydrophobic and spacer positions. We divided these by total number of hydrophobic positions (4 times the number of NESs) and the number of spacer positions. This gave the probability parameters of each amino acid at the two types of positions for the NoLogo model. The second is the spacer length probability model, which was obtained by counting the number of spacers of each size at each position separately and then dividing by the total number of NESs. We smoothed all these estimates by adding a pseudocount of 1 at each amino acid position or spacer size.

To predict NESs using the NoLogo model, we use the likelihood ratio score (described above formula 4). The log-likelihood ratio score for NoLogo was evaluated as follows.

8 $$ \mathrm{The}\ \mathrm{llr}=\log \frac{\mathrm{P}\left(\mathrm{X}|\mathrm{No}\ \mathrm{Logo}\right)}{\mathrm{P}\left(\mathrm{X}|\mathrm{bg}\right)}=\log \frac{\sum_CP(C){\prod}_{i\in {\Phi}_C}{p}_{\Phi}\left({X}_i\right){\prod}_{j\in {S}_C}{p}_S\left({X}_j\right){\prod}_{k= length(C)+1}^{k=w}P\left({X}_k| bg\right)}{\sum_C\frac{1}{27}{\prod}_{i=1}^wP\left({X}_i| bg\right)} $$

In this expression, *X* is a sequence of length *w*, the sums are over all 27 configurations, *C*. *P*(*C*) is the probability of the configuration under the NES model as described above. 1/27 is a uniform distribution over the configurations, reflecting the assumption that under the background model, the three spacer lengths have equal probability of 1/3 at each position, such that all configurations are equally likely. *P*(*X*_*i*_| *bg*) is the probability of residues under the background distribution, which we estimated from the entire *S. cerevisiae* proteome. *p*_Φ_ and *p*_S_ are the probability models for the hydrophobic and spacer amino acid positions as described above. For example, a 3–2-1 NES, *C* = (3,2,1), has 4 hydrophobic residues and 6 spacer residues, for a total length of 10 residues. We indicate the length of the NES using *length*(*C*). Since the length varies depending on the configuration, and 13 residues is the maximum length, *C* = (3,3,3), we compute the probability of 13 residues, regardless of the length of the configuration. Therefore, we use *k* to index additional amino acid positions beyond the length of *C*, and we compute their probability using the background model.

### Prediction performance evaluation

To assess prediction performance for the algorithms we employed ROC curve analysis. The approach is similar to that in previous work [36]. For the true negative set, we calculated the total length of proteins in the test set in amino acids and subtracted the total length of amino acids that constitute all the NESs. There were 279 NESs in NESdb (lacking *S. cerevisiae* training set proteins); their total combined amino acid length is 2989 amino acids and the length of their protein sequences combined is 145,512 amino acids. Thus, the actual amino acids that are not part of the NESs in the test set proteins is 145,512–2989 = 142,523 amino acids. This constitutes the true negative set for the ROC curve analysis.

To calculate a proteome-wide level false positive rate, in order to evaluate the performance of an algorithm’s NES prediction rates, we reasoned as follows. In our approximation of the expected true positive and negative set, we first calculated the approximate number of proteins in the proteome expected to harbor NESs. For this we used the estimate of approximately 700 proteins in *S. cerevisiae* considered to be direct or indirect substrates of Crm1 [37]. We believe this to be a liberal approximation of the true positive set. Thus, the fraction of the *S. cerevisiae* proteome that could have Crm1 dependent NESs is 700/5917, which is approximately 0.12. At a per amino acid residue level it would be the 0.12 fraction divided by the average protein length in *S. cerevisiae* (495 amino acids), which yields 0.000242. This value represents the expected rate of NESs in the proteome at per residue level and therefore the rate at which we evaluate the predictive power of the algorithms. We make the assumption that this rate also applies to other species to simplify the calculation. Others [6, 18] have noted that engineering an algorithm to predict NESs at a proteome-wide level is unfeasible and consequently the algorithms assume the query protein sequences contain an NES.

The following describes the heuristics we applied to parse the True positives and False positives in the NES predictions. Our collection of true positive NESs and their amino acid coordinates in the test set were taken from [6]. For NoLogo and PSSM models, we collected all candidates that scored greater than 0 in log-likelihood ratio score. For GLAM2 we set the limit to top 18,063 scoring predictions, which is the number of predictions that is in the range of predictions made by NoLogo and PSSM models. Any candidate NES predicted whose amino acid coordinates in the protein falls outside the coordinates of the true positives is considered a false positive. To remove redundant NES predictions, which overlap each other, for each algorithm, we employed the following criteria. For predictions that overlapped with a positive true NES we required at least one amino acid overlap with the coordinates provided in Additional file 1: Table S1 of [6]. Once all the predictions that overlap with the true positive NES are collected then the candidate that scored the highest in log-likelihood ratio is chosen as the representative NES for the true positive NES. Similarly, for predictions that fall outside the region of true positive NES, we categorized as false positives and tiled all predictions that overlap with each other by at least one amino acid and selected the highest scoring segment as representative of that region encompassed by that set of tiled predictions.

We performed the GLAM2 analysis using a locally installed command line version of GLAM2 that is available at(http://meme-suite.org/tools/glam2) instead of the web based version due to input and output capacity limitations.

Wregex [19] available at http://ehubio.ehu.eus/wregex/faces/search.xhtml (as of 09/05/2017) was used under Relaxed configuration setting with the following regular expression ([LIVMFAWY])(.{2,3})([LIVMFAWY])([^P]{2,3})([LIVMFAWY])([^P])([LIVMFAWY]). Regular expression relaxed and PSSM computed with ValidNES, DUBs and Ligases without assay scores.

We ran NESmapper [18] locally using a perl script available at https://sourceforge.net/projects/nesmapper/ (as of 09/05/2017) and was used with “NES_profiles_trained” setting and its output was processed similarly to NoLogo’s output.

LocNES analysis was performed using the web version available at http://prodata.swmed.edu/LocNES/LocNES.php (as of 09/05/2017). We note that the training set available for LocNES was more expansive than all the other algorithms and by far compared to NoLogo.

The clustered heat map data were generated using ComplexHeatmap package [38], using Euclidian distance and average linkage clustering methods. To evaluate whether the differences in proportion of true positive to false negative between two algorithms at specific false positive rates were statistically significant, we performed Fisher’s exact test of independence in the R statistical software environment [39] and RStudio [40] with graphical packages [41].

## Additional files

Additional file 1: **Table S1.** Evidence and references for characterized yeast NESs used in this study. (XLSX 38 kb)

Additional file 2: **Figure S1.** Analysis of 35 *S. cerevisiae* NESs manually aligned based on their hydrophobic positions for the NoLogo model. (PDF 100 kb)

Additional file 3: Derivation of the E-M algorithm to estimate parameters for the NoLogo model from unaligned sequences. (DOCX 21 kb)

Additional file 4: **Figure S2.** NoLogo model parameters derived through E-M algorithm. The top panel consists of a bar graph depicting the hydrophobic position and spacer position probabilities. The bottom panel is a bar graph of the spacer length probabilities. (PDF 71 kb)

Additional file 5: **Figure S3.** An ROC curve analysis of prediction performance. a) NoLogo trained on a subset of the 35ScNESs sampled randomly as 5, 10, 15, 20, 25, 30 or 35 NESs and predictions made on the NESdb proteins. PSSM’s, trained on 35ScNESs, performance is included for comparison. b) Similarity in NoLogo prediction performance using parameters derived from hand alignment or from E-M algorithm. (PDF 1008 kb)

Additional file 6: **Figure S4.** Some sample motifs discovered by GLAM2 in the 25 amino acids around the NESs in the 35ScNES set. a) This sequence logo depicts the best motif discovered by GLAM2 among 10 runs. b) Another sequence logo of a motif with intermediate score. c) One of the lowest scoring motifs that is subsequently used to scan the NESdb proteins for NES motifs. (PDF 33 kb)

Additional file 7: **Figure S5.** LocNES consistently performs better than all predictive algorithms. Performance was assayed on Sacchromyces cerevisiae specific NESs. These Yeast NESs consisted of 19-test NESs that did not overlap with training set for LocNES and 15-training set NESs that overlapped with LocNES training set. (PDF 134 kb)

Additional file 8: **Figure S6.** This is the heat map generated from spacer configuration assignment probabilities of NESdb NESs using NoLogo with parameters estimated with the EM algorithm. (PDF 134 kb)

Additional file 9: **Figure S7.** NoLogo predictive performance not affected upon restriction to using only the 10 canonical classes as opposed to 27 possible spacer configuration categories. ROC curve analysis of NoLogo using all 27 possible spacer configurations (green line curve) vs 10 possible spacer configurations (purple line curve). (PDF 123 kb)

Additional file 10: **Figure S8.** NoLogo predictive performance not affected upon restriction to using only the 10 canonical classes as opposed to 27 possible spacer configuration categories. a) Posterior probabilities of NES configurations, using only the 10 canonical classes, are indicated in a heat map, where black corresponds to higher probabilities. Two hundred seventy-nine NESs have been clustered based on similarity, while configurations are ordered from least to most populated (top to bottom). Clusters corresponding to previously known classes of NESs are indicated as blue lines, while NESs exhibiting multiple classes are indicated as red boxes. b) The sample NES from Fig. 5 that is re-analyzed using settings that only allow 10 canonical spacer configurations. In Additional file 10: Figure S8b, this NES adopts 2–2-1 configuration whereas in Fig. 5b it exhibited 2–2-3 configuration. c) displays the posterior probability distribution of spacer configurations for an NES that exhibited up to 4 class membership with equal probabilities in Fig. 5c but now appears to be described by the 2–2-1 spacer configuration, solely, when NoLogo is restricted to just the 10 canonical spacer configuration classes. d) displays the posterior probability distribution of spacer configurations for an NES that exhibits up to 4 class membership, when NoLogo is restricted to just the 10 canonical spacer configuration classes. (PDF 938 kb)

Additional file 11: **Table S2.** Background proteome amino acid frequencies used in this study. (DOCX 81 kb)

## Abbreviations

17hcScNES: 17 high confidence *S. cerevisiae* nuclear export signal
35hcScNES: 35 high confidence *S. cerevisiae* nuclear export signal
E-M: Expectation maximization
FPR: False positive rate
HMM: Hidden markov model
llr: Log-likelihood ratio
NESs: Nuclear export signals
NLSs: Nuclear localization signals
PSSM: Position specific scoring matrix
PWM: Position weight matrix
SVM: Support vector machines
TPR: True positive rate

## References

[1] Stormo GD (2000). DNA binding sites: representation and discovery. Bioinformatics.

[2] Schneider TD, Stephens RM (1990). Sequence logos: a new way to display consensus sequences. Nucleic Acids Res.

[3] Crooks GE, Hon G, Chandonia J-M, Brenner SE (2004). WebLogo: a sequence logo generator. Genome Res.

[4] Wen W, Meinkoth JL, Tsien RY, Taylor SS (1995). Identification of a signal for rapid export of proteins from the nucleus. Cell.

[5] Fischer U, Huber J, Boelens WC, Mattaj IW, Lührmann R (1995). The HIV-1 rev activation domain is a nuclear export signal that accesses an export pathway used by specific cellular RNAs. Cell.

[6] Xu D, Marquis K, Pei J, Fu S-C, Cağatay T, Grishin NV (2015). LocNES: a computational tool for locating classical NESs in CRM1 cargo proteins. Bioinformatics.

[7] Fornerod M, Ohno M, Yoshida M, Mattaj IW (1997). CRM1 is an export receptor for leucine-rich nuclear export signals. Cell.

[8] Stade K, Ford CS, Guthrie C, Weis K (1997). Exportin 1 (Crm1p) is an essential nuclear export factor. Cell.

[9] Fukuda M, Asano S, Nakamura T, Adachi M, Yoshida M, Yanagida M (1997). CRM1 is responsible for intracellular transport mediated by the nuclear export signal. Nature.

[10] Ossareh-Nazari B, Bachelerie F, Dargemont C (1997). Evidence for a role of CRM1 in signal-mediated nuclear protein export. Science.

[11] Neville M, Stutz F, Lee L, Davis LI, Rosbash M (1997). The importin-beta family member Crm1p bridges the interaction between rev and the nuclear pore complex during nuclear export. Curr Biol.

[12] Kosugi S, Hasebe M, Tomita M, Yanagawa H (2008). Nuclear export signal consensus sequences defined using a localization-based yeast selection system. Traffic.

[13] la Cour T, Kiemer L, Mølgaard A, Gupta R, Skriver K, Brunak S (2004). Analysis and prediction of leucine-rich nuclear export signals. Protein Eng Des Sel.

[14] Monecke T, Güttler T, Neumann P, Dickmanns A, Görlich D, Ficner R (2009). Crystal structure of the nuclear export receptor CRM1 in complex with Snurportin1 and RanGTP. Science.

[15] Güttler T, Madl T, Neumann P, Deichsel D, Corsini L, Monecke T (2010). NES consensus redefined by structures of PKI-type and rev-type nuclear export signals bound to CRM1. Nat Struct Mol Biol.

[16] Fung HYJ, Fu S-C, Brautigam CA, Chook YM (2015). Structural determinants of nuclear export signal orientation in binding to exportin CRM1. elife.

[17] Fu S-C, Imai K, Horton P (2011). Prediction of leucine-rich nuclear export signal containing proteins with NESsential. Nucleic Acids Res.

[18] Kosugi S, Yanagawa H, Terauchi R, Tabata S (2014). NESmapper: accurate prediction of leucine-rich nuclear export signals using activity-based profiles. PLoS Comput Biol.

[19] Prieto G, Fullaondo A, Rodriguez JA (2014). Prediction of nuclear export signals using weighted regular expressions (Wregex). Bioinformatics.

[20] la Cour T, Gupta R, Rapacki K, Skriver K, Poulsen FM, Brunak S (2003). NESbase version 1.0: a database of nuclear export signals. Nucleic Acids Res.

[21] Fu S-C, Huang H-C, Horton P, Juan H-F (2013). ValidNESs: a database of validated leucine-rich nuclear export signals. Nucleic Acids Res.

[22] Xu D, Grishin NV, Chook YM (2012). NESdb: a database of NES-containing CRM1 cargoes. Mol Biol Cell.

[23] Fung HYJ, Fu S-C, Chook YM (2017). Nuclear export receptor CRM1 recognizes diverse conformations in nuclear export signals. elife.

[24] Xu D, Farmer A, Collett G, Grishin NV, Chook YM (2012). Sequence and structural analyses of nuclear export signals in the NESdb database. Mol Biol Cell.

[25] Tompa P, Davey NE, Gibson TJ, Babu MM (2014). A million peptide motifs for the molecular biologist. Mol Cell.

[26] Obenauer JC, Cantley LC, Yaffe MB (2003). Scansite 2.0: Proteome-wide prediction of cell signaling interactions using short sequence motifs. Nucleic Acids Res.

[27] Bailey TL, Elkan C (1994). Fitting a mixture model by expectation maximization to discover motifs in biopolymers. Proc Int Conf Intell Syst Mol Biol.

[28] Moses A, Sinha S, Edwards D, Stajich J, Hansen D (2009). Regulatory motif analysis. Bioinforma. - tools Appl. [internet].

[29] Durbin R, Eddy SR, Krogh A, Mitchison G. Biological sequence analysis: probabilistic models of proteins and nucleic acids. Cambridge: Cambridge University Press; 1998.

[30] Frith MC, Saunders NFW, Kobe B, Bailey TL (2008). Discovering sequence motifs with arbitrary insertions and deletions. PLoS Comput Biol.

[31] Dong X, Biswas A, Süel KE, Jackson LK, Martinez R, Gu H (2009). Structural basis for leucine-rich nuclear export signal recognition by CRM1. Nature.

[32] Sharma R, Raduly Z, Miskei M, Fuxreiter M (2015). Fuzzy complexes: specific binding without complete folding. FEBS Lett.

[33] Miskei M, Antal C, Fuxreiter M (2017). FuzDB: database of fuzzy complexes, a tool to develop stochastic structure-function relationships for protein complexes and higher-order assemblies. Nucleic Acids Res.

[34] Jones DT, Cozzetto D (2015). DISOPRED3: precise disordered region predictions with annotated protein-binding activity. Bioinformatics.

[35] Kutay U, Güttinger S (2005). Leucine-rich nuclear-export signals: born to be weak. Trends Cell Biol.

[36] Nguyen Ba AN, Pogoutse A, Provart N, Moses AM (2009). NLStradamus: a simple hidden Markov model for nuclear localization signal prediction. BMC Bioinformatics.

[37] Kırlı K, Karaca S, Dehne HJ, Samwer M, Pan KT, Lenz C, et al. A deep proteomics perspective on CRM1-mediated nuclear export and nucleocytoplasmic partitioning. eLife. 2015; [cited 2017 May 3];4. Available from: http://www.ncbi.nlm.nih.gov/pmc/articles/PMC4764573/10.7554/eLife.11466 PMC4764573 26673895

[38] Gu Z, Eils R, Schlesner M (2016). Complex heatmaps reveal patterns and correlations in multidimensional genomic data. Bioinformatics.

[39] R Core Team (2016). R: a language and environment for statistical computing [internet].

[40] Team R (2016). RStudio: integrated development for R. RStudio, Inc. [internet].

[41] Wickham H (2009). ggplot2: elegant graphics for data analysis [internet].

