# Supplementary material for: NoLogo: a new statistical model highlights the diversity and suggests new classes of Crm1-dependent nuclear export signals
Source: BMC Bioinformatics. 2018 Feb 27;19:65. doi: 10.1186/s12859-018-2076-7 (PMC5828312; doi:10.1186/s12859-018-2076-7)
Supplement: Supplementary file 2 — Figure S1. Analysis of 35 S. cerevisiae NESs manually aligned based on their hydrophobic positions for the NoLogo model. (PDF 100 kb) [file 12859_2018_2076_MOESM2_ESM.pdf]

	Φ_1	Spacer 1			Φ_2	Spacer 2			Φ_3	Spacer 3			Φ_4	Spacer Configuration		
YLR131C_ACE2NES_136	L	S			I	S	S		L	Q	Q	S	I	1	2	3
YFL039C_ACT1NES_171	L	P	H	A	I	L	R		I	D			L	3	2	1
YFL039C_ACT1NES_212	I	K	E	K	L	C	Y		V	A			L	3	2	1
YGL071W_AFT1NES_100	I	H			L	D	P		V	P	N		F	1	2	2
YPR093C_ASR1NES_103	L	S	E	R	L	R	G	T	L	V			M	3	3	1
YPR093C_ASR1NES_125	G	D	T	D	V	S	R		L	S			L	3	2	1
YLR074C_BUD20NES_18	L	D	L		I	Y	N	D	L	S	T	K	E	2	3	3
YPR119W_CLB2NES_297	L	V	Q		L	D	K		L	Q			L	2	2	1
YMR232W_FUS2NES_91	F	Q	N		V	V	R		L	N			L	2	2	1
YDL207W_GLE1NES_351	L	P			L	G	K		L	T			L	1	2	1
YER040W_GLN3NES_64	L	E	A		L	P	D	D	L	Y			F	2	3	1
YPL015C_HST2NES_308	I	V	H	D	L	E	N		L	S			L	3	2	1
YGL253W_HXK2NES_310	L	G	E		I	L	R		L	A			L	2	2	1
YGL253W_HXK2NES_23	L	M	Q	Q	I	E	N		F	E	K		I	3	2	2
YLR347C_KAP95NES_55	L	E	G	R	I	L	A	A	L	T			L	3	3	1
YDR499W_LCD1NES_667	V	D	S		L	H	D		L	T			I	2	2	1
YKL143W_LTV1NES_451	L	E	K		V	T	N	T	L	S	S		L	2	3	2
YEL032W_MCM3NES_834	L	Q	R	R	L	Q			L	G			L	3	1	1
YOL111C_MDY2NES_117	I	K			L	L			L	K	G	K	V	1	1	3
YKR048C_NAP1NES_88	L	P	K	N	V	K	E	K	L	L	S		L	3	3	2
YHR170W_NMD3NES_491	I	N			I	D	E		L	L	D	E	L	1	2	3
YER165W_PAB1NES_9	A	E	Q		L	E	N		L	N			I	2	2	1
YIR006C_PAN1NES_1147	L	Q	E	E	L	K	R		L	K			L	3	2	1
YJL128C_PBS2NES_1	M	E	D	K	F	A	N		L	S			L	3	2	1
YBL105C_PKC1NES_54	L	E	D	S	L	K	K		L	R			L	3	2	1
YKL012W_PRP40NES_274	L	K	E		L	R	E	Y	L	N	G		I	2	3	2
YKL012W_PRP40NES_340	L	Q	N	K	L	N	E		L	R			L	3	2	1
YMR235C_RNA1NES_320	L	P	E		L	E	K		L	E			I	2	2	1
YMR235C_RNA1NES_343	L	Q	S	K	F	D	D		L	E			V	3	2	1
YER125W_RSP5NES_687	L	I	G	G	I	A	E		I	D			I	3	2	1
YOR329C_SCD5NES_851	L	G	N		L	Q	S		L	Q	Q	Q	V	2	2	3
YAL047C_SPC72NES_418	L	E	K	Q	I	N	D		L	Q			I	3	2	1
YDL229W_SSB1NES_578	L	S	D	A	L	A	A		L	Q			I	3	2	1
YJL187C_SWE1NES_67	L	N			L	S			L	S	N	T	A	1	1	3
YML007W_YAP1NES_614	I	D			V	D	G		L	C	S	E	L	1	2	3
