# Supplementary material for: NoLogo: a new statistical model highlights the diversity and suggests new classes of Crm1-dependent nuclear export signals
Source: BMC Bioinformatics. 2018 Feb 27;19:65. doi: 10.1186/s12859-018-2076-7 (PMC5828312; doi:10.1186/s12859-018-2076-7)
Supplement: Supplementary file 3 — Derivation of the E-M algorithm to estimate parameters for the NoLogo model from unaligned sequences. (DOCX 21 kb) [file 12859_2018_2076_MOESM3_ESM.docx]

For a collection of *N* sequences, *X*, we assume that the motif could be at any subsequence of *w* residues. If the motif does not occur at a given starting position, *i*, then we assume that subsequence was generated by a background model. This gives a two-component mixture with likelihood, *L*:

$$L=\prod_{n \epsilon sequences} \sum_{i \epsilon starts} P\left( start at i \right)P\left( X_{n,i..i+w} | nologo \right)+\left( 1-P\left( start at i \right) \right)P\left( X_{n,i..i+w} | g \right)$$

We let *P*(*start at i*) = *π*, and assume it is uniform over the sequence: we don’t have any knowledge about where we expect the NES to start. *g* represents a background sequence model that gives the probability of each residue in the absence of an NES.

$$P\left( X_{n,i..i+w} | nologo \right)=\sum_{C \epsilon config.s} P\left( C | p_{C} \right)P(X_{n,i..i+w}|C,p_{\Phi},p_{S},g)$$

The sum is over the 27 possible configurations, *C*, each weighted by their probability *P*(*C*|$p_{C}$), where $p_{C}$ is a matrix of spacer length probabilities, and *C* = (*C_1_*, *C_2_*, *C_3_*) indicates the lengths of the spacers in configuration *C*, such that *C_ab_* = 1 if the *a*-th spacer in configuration *C* has length *b*, and 0 if it doesn’t. The probability of the residues given a configuration is determined only by the residue probability models, *p*, for the hydrophobic positions, $\Phi$, the spacers *S*, and the background model, *g*. Expanding out the probability model, we have

$$P\left( X_{n,i..i+w} | nologo \right)=\sum_{C \epsilon config.s} \prod_{a,b\epsilon1,2,3} {p_{C_{ab}}}^{C_{ab}}\prod_{k=i}^{k=i+length(C)} {P(X_{nk}|p_{\Phi})}^{H(C,k-i)}{P(X_{nk}|p_{S})}^{1-H(C,k-i)}\prod_{j=i+length\left( C \right)+1}^{j=i+w} P(X_{nj}|g)$$

Where *k* indexes the residues in the NES, while *j* indexes the residues in the “tail” of background residues needed to make the total length of the segment add up to *w*. *H* is a known indicator variable that, for each configuration, *C*, is 1 if position *k* is hydrophobic and 0 if spacer. The length of configuration *C* is $length\left( C \right)=4+\sum_{ab} bC_{ab}$, where the 4 is from the hydrophobic residues. The categorical models for the amino acid residues have the forms

$$P\left( X_{ni} | p_{\Phi} \right)=\prod_{r\epsilon A,C,D,E\ldots} {p_{\Phi r}}^{X_{nir}}$$

$$P\left( X_{ni} | p_{S} \right)=\prod_{r\epsilon A,C,D,E\ldots} {p_{Sr}}^{X_{nir}}$$

where *r* indexes the amino acid residues. The background model, *g*, is similar.

The log likelihood is then

$$\log L=\sum_{n} \log\left[ \sum_{i} \pi\sum_{C} P\left( C | p_{C} \right)P(X_{n,i..i+w}|C,p_{\Phi},p_{S},g)+(1-\pi)P(X_{n,i..i+w}|g) \right]$$

For EM, we use the complete likelihood.

$$L_{compl.}=\prod_{n} \prod_{i} \left[ \pi\prod_{C} \left[ P\left( C | p_{C} \right)P(X_{n,i..i+w}|C,p_{\Phi},p_{S},g) \right]^{V_{nic}} \right]^{Z_{ni}}\left[ (1-\pi)P(X_{n,i..i+w}|g) \right]^{1-Z_{ni}}$$

Where *Z* is an unobserved indicator variable that equals 1 at the “true” start, and 0 otherwise, and *V* is an unobserved indicator variable that equals 1 in the “true” configuration and 0 for all others. We can now take logs and expectations to give:

$$\left\langle\log L_{compl.} \right\rangle=\sum_{n} \left[ \sum_{i} \left\langle Z_{ni} \right\rangle\sum_{C} \left\langle V_{niC} \right\rangle\left[ \log P\left( C | p_{C} \right)+\log P(X_{n,i..i+w}|C,p_{\Phi},p_{S},g) \right]+const. \right]$$

In the E step, we fill in the unobserved *Z* and *V* indicator variables with their expectations, which are given by the posterior probabilities of the positive outcome. These are calculated using the formulas given in the text, e.g.,$\left\langle V_{niC} \right\rangle=P(C|X_{n,i..i+w})$ . To derive the M step, we maximize by taking the derivatives with respect to the parameters and setting to 0. Since we assume the background probabilities and the probability that the motif starts at a given position, *π*, are constant, several terms do not depend on the parameters, and we can lump them together in a constant that will vanish. For example,

$$\frac{\partial\left\langle\log L_{compl.} \right\rangle}{\partial p_{Cab}}=\sum_{n} \left[ \sum_{i} \left\langle Z_{ni} \right\rangle\sum_{C} \left\langle V_{niC} \right\rangle\frac{\partial\log P\left( C | p_{C} \right)}{\partial p_{Cab}} \right]+\frac{\partial}{\partial p_{Cab}}\gamma\left( 1-\sum_{b} p_{Cab} \right)=0$$

Where we have added a lagrange multiplier$\gamma$ to ensure the $p_{C}$ add up to 1 at each spacer position.

$$\frac{\partial\left\langle\log L_{compl.} \right\rangle}{\partial p_{Cab}}=\sum_{n} \left[ \sum_{i} \left\langle Z_{ni} \right\rangle\sum_{C} \left\langle V_{niC} \right\rangle\frac{C_{ab}}{p_{Cab}} \right]-\gamma=0$$

Solving this and substituting to get the lagrange multiplier gives

$$p_{Cab}=\frac{1}{N}\sum_{n} \left[ \sum_{i} \left\langle Z_{ni} \right\rangle\sum_{c} \left\langle V_{niC} \right\rangle C_{ab} \right]$$

Where *N* is the total number of sequences. Similar calculations give the estimates of the frequency for each residue, *r*, in the hydrophobic positions:

$$p_{\Phi r}=\frac{1}{4N}\sum_{n} \sum_{i} \left\langle Z_{ni} \right\rangle\sum_{C} \left\langle V_{niC} \right\rangle\sum_{k=i}^{k=i+length(C)} H(C,k-i)X_{nkr}$$

Where the denominator comes from the constraint that there are exactly 4 hydrophobic positions in each motif. For the spacer positions, we have to estimate how many there are. We have for each residue, *r*:

$$p_{Sr}=\frac{1}{\sum_{n} \sum_{i} \left\langle Z_{ni} \right\rangle\sum_{C} \left\langle V_{niC} \right\rangle\sum_{k} \left[ 1-H\left( C,k-i \right) \right]}\sum_{n} \sum_{i} \left\langle Z_{ni} \right\rangle\sum_{C} \left\langle V_{niC} \right\rangle\sum_{k=i}^{i+length(C)} \left[ 1-H\left( C,k-i \right) \right]X_{nkr}$$

These formulas complete the M-step.
