# Supplementary figures and images for: NoLogo: a new statistical model highlights the diversity and suggests new classes of Crm1-dependent nuclear export signals
Source: BMC Bioinformatics. 2018 Feb 27;19:65. doi: 10.1186/s12859-018-2076-7 (PMC5828312; doi:10.1186/s12859-018-2076-7)

**a**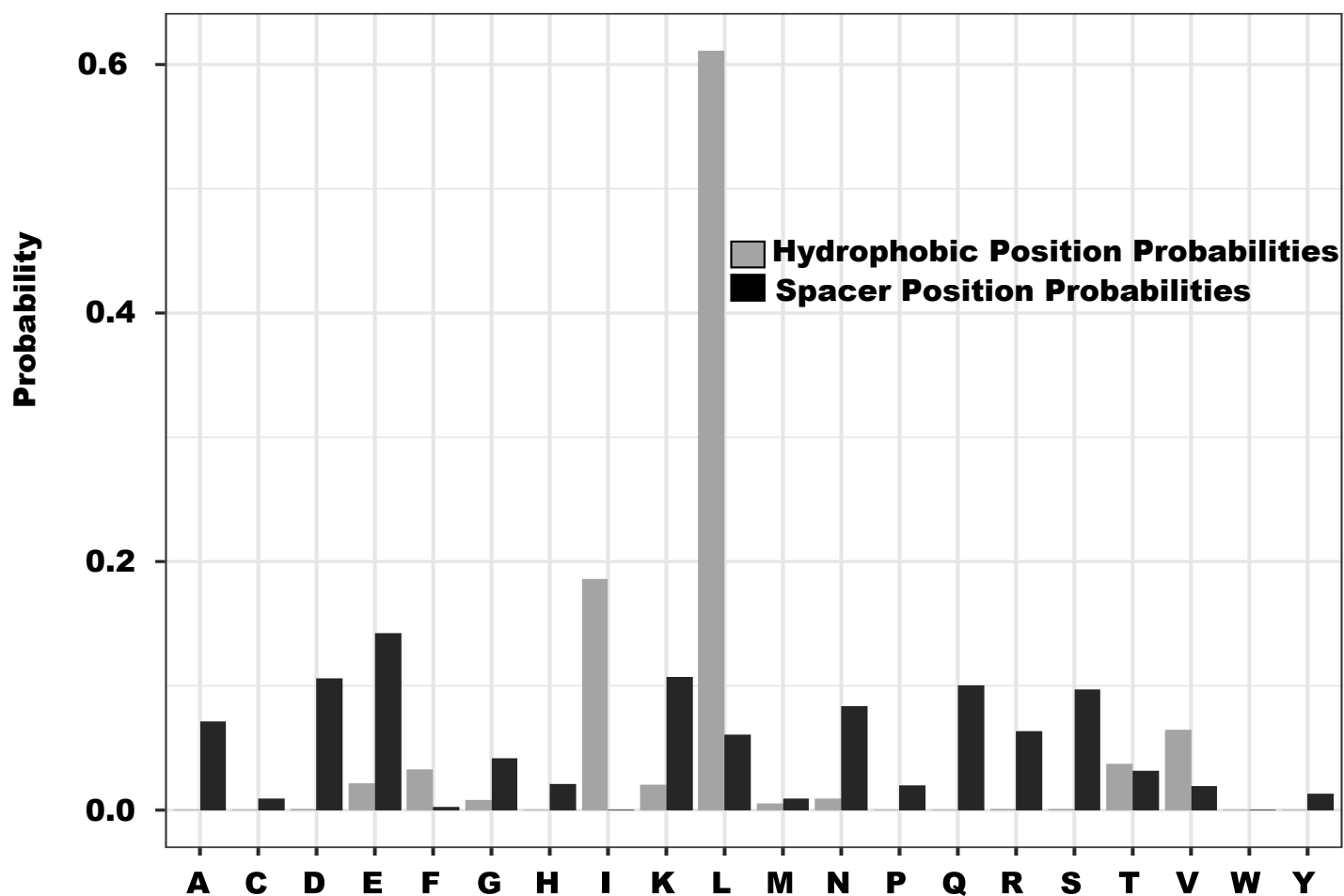**b**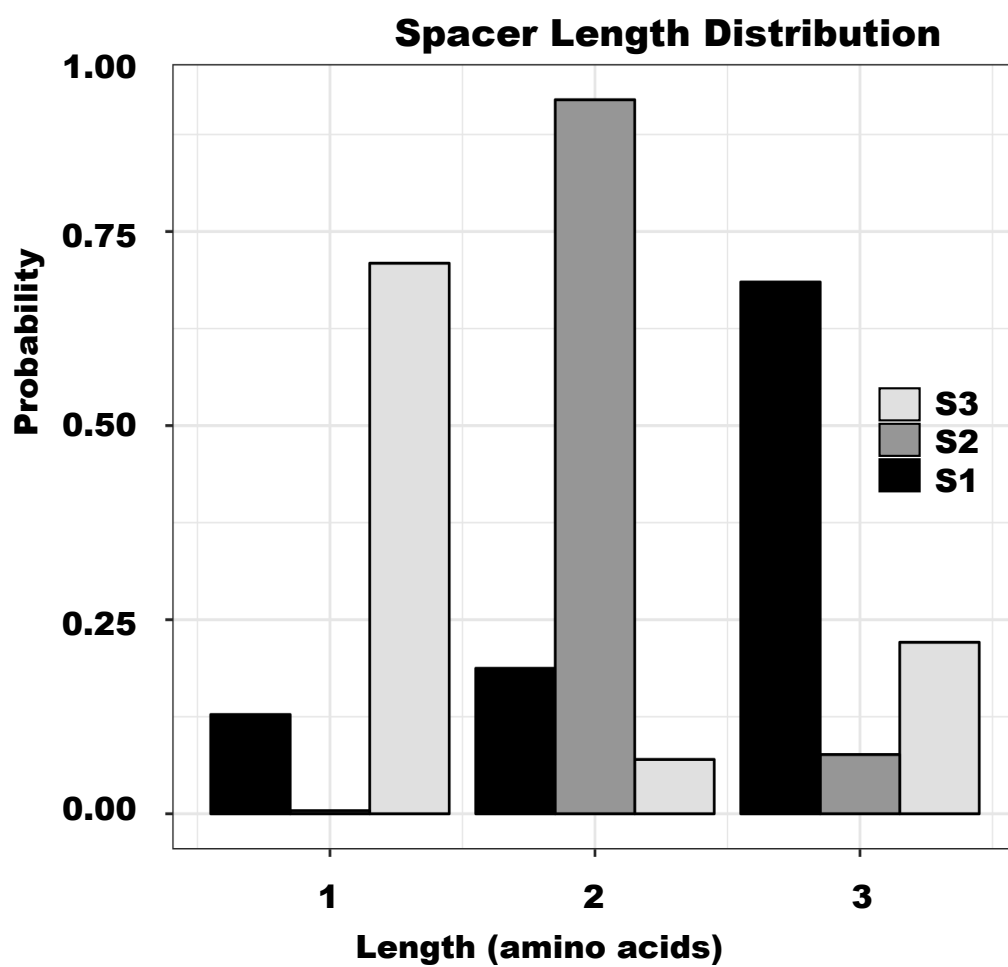

Supplement: Supplementary file 4 — Figure S2. NoLogo model parameters derived through E-M algorithm. The top panel consists of a bar graph depicting the hydrophobic position and spacer position probabilities. The bottom panel is a bar graph of the spacer length probabilities. (PDF 71 kb) [file 12859_2018_2076_MOESM4_ESM.pdf]

**a**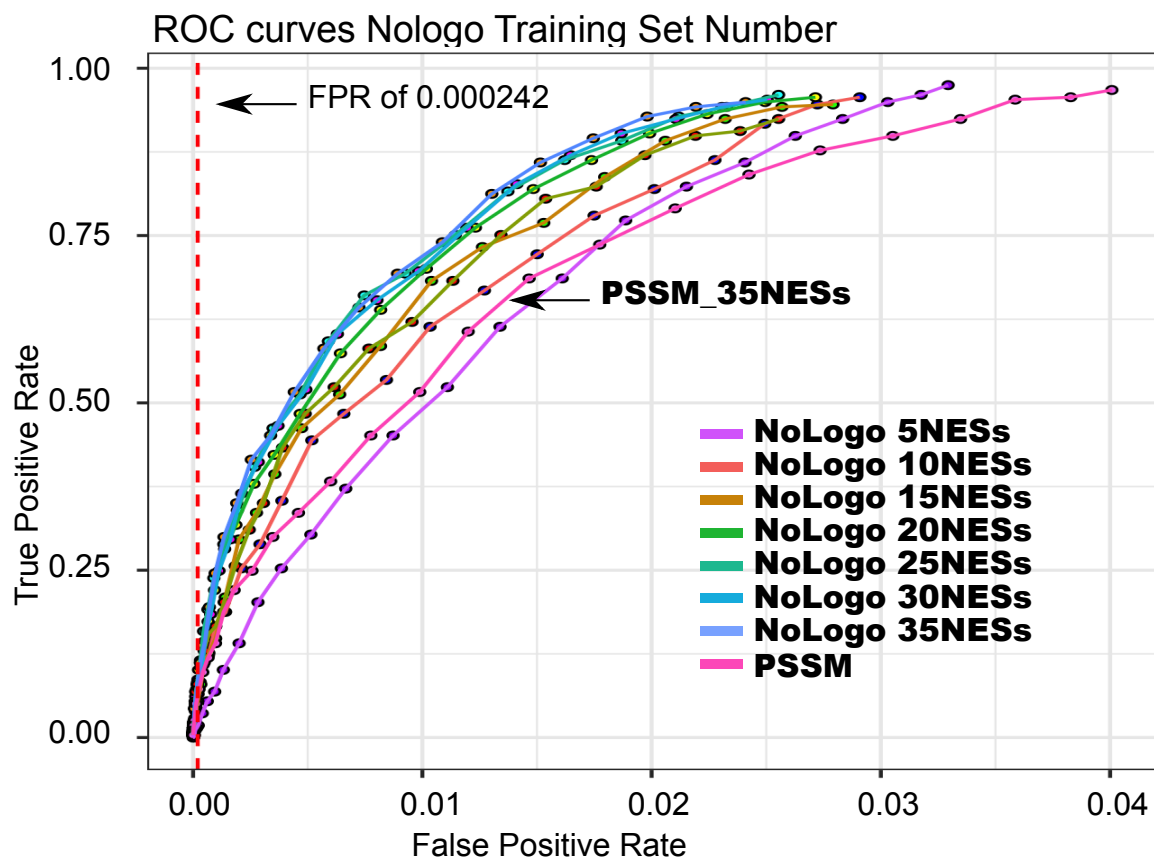**b**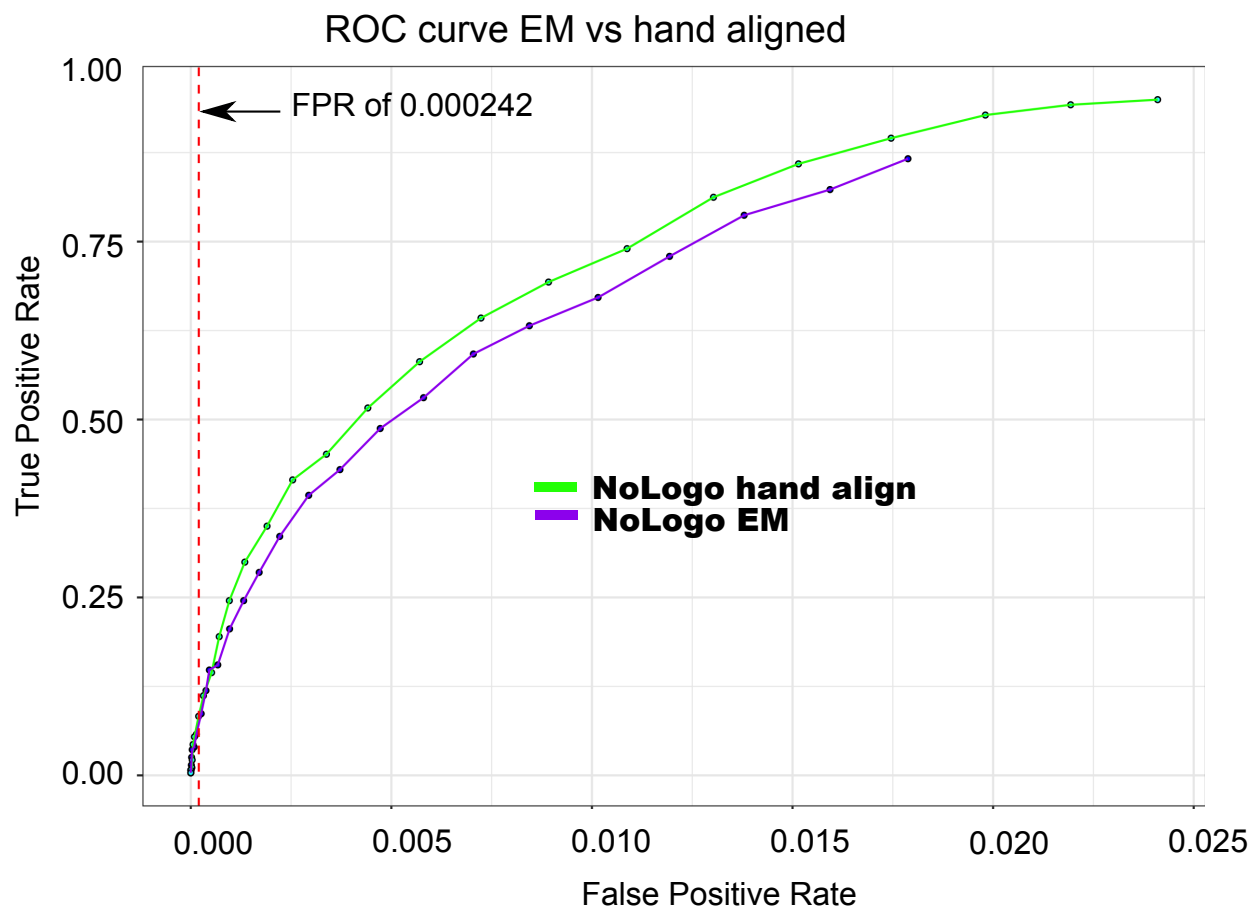

Supplement: Supplementary file 5 — Figure S3. An ROC curve analysis of prediction performance. a) NoLogo trained on a subset of the 35ScNESs sampled randomly as 5, 10, 15, 20, 25, 30 or 35 NESs and predictions made on the NESdb proteins. PSSM’s, trained on 35ScNESs, performance is included for comparison. b) Similarity in NoLogo prediction performance using parameters derived from hand alignment or from E-M algorithm. (PDF 1008 kb) [file 12859_2018_2076_MOESM5_ESM.pdf]

a

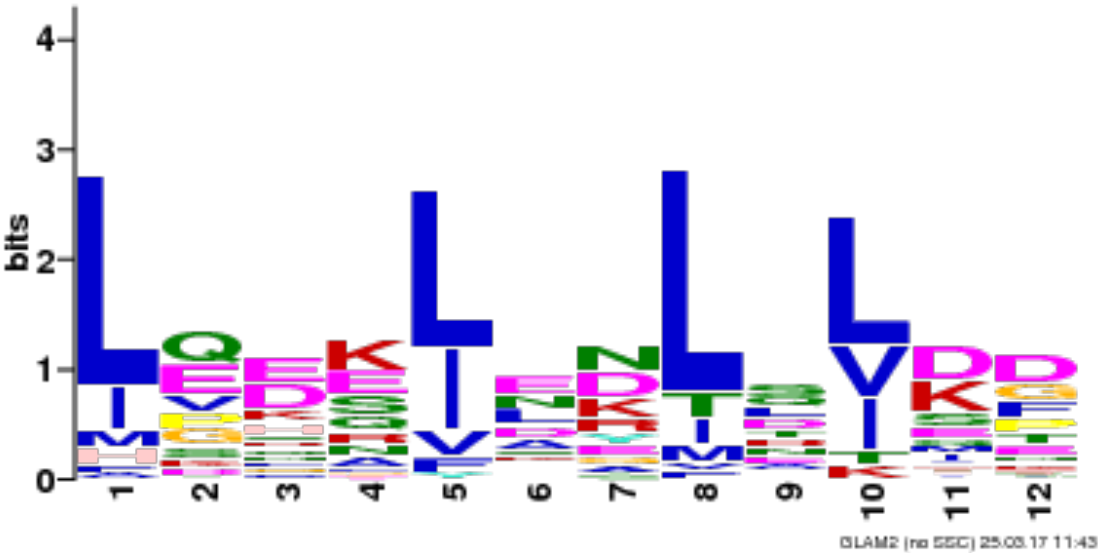

b

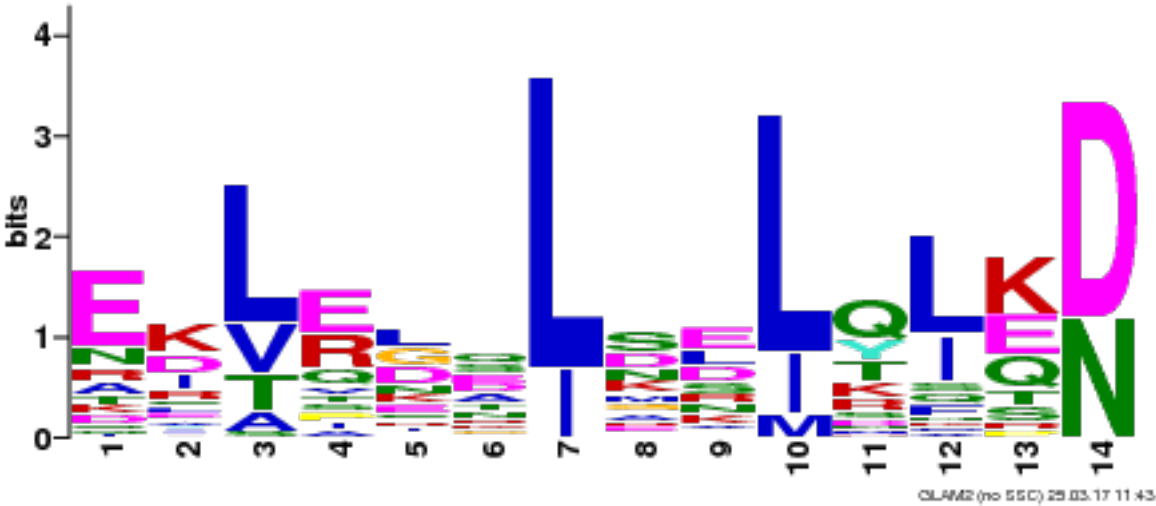

c

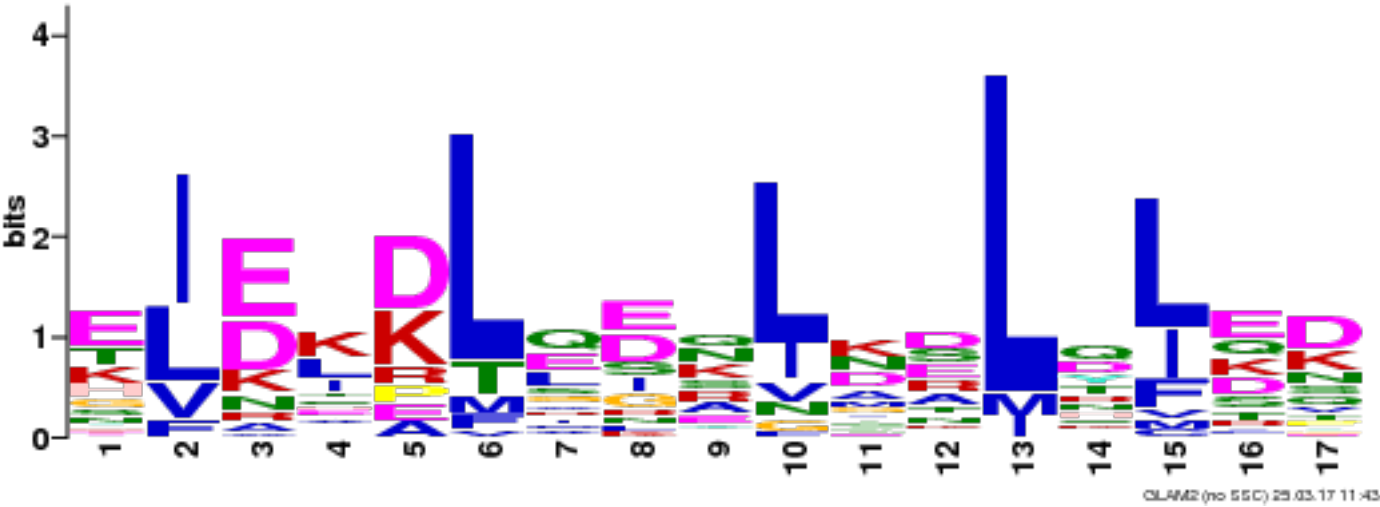

Supplement: Supplementary file 6 — Figure S4. Some sample motifs discovered by GLAM2 in the 25 amino acids around the NESs in the 35ScNES set. a) This sequence logo depicts the best motif discovered by GLAM2 among 10 runs. b) Another sequence logo of a motif with intermediate score. c) One of the lowest scoring motifs that is subsequently used to scan the NESdb proteins for NES motifs. (PDF 33 kb) [file 12859_2018_2076_MOESM6_ESM.pdf]

ROC curve NoLogo vs Wregex vs LocNES vs NESmapper

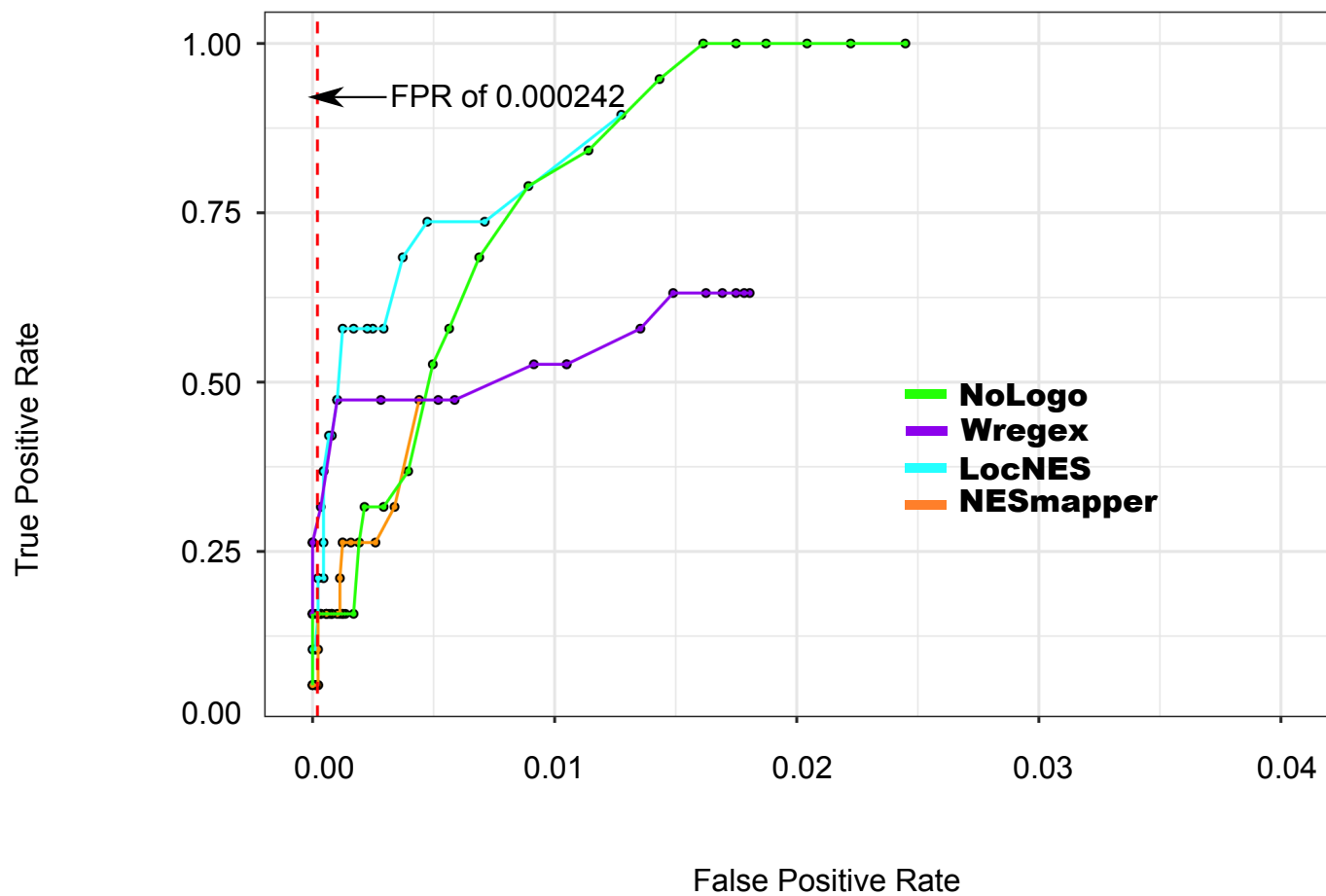

Supplement: Supplementary file 7 — Figure S5. LocNES consistently performs better than all predictive algorithms. Performance was assayed on Sacchromyces cerevisiae specific NESs. These Yeast NESs consisted of 19-test NESs that did not overlap with training set for LocNES and 15-training set NESs that overlapped with LocNES training set. (PDF 134 kb) [file 12859_2018_2076_MOESM7_ESM.pdf]

**NESs**

**Spacer Configurations**

3-1-2  
2-1-2  
1-1-3  
1-1-2  
2-1-3  
3-1-3  
1-3-2  
3-3-2  
2-1-1  
2-3-3  
3-1-1  
1-3-3  
2-3-2  
1-1-1  
2-2-2  
1-2-2  
2-3-1  
3-3-3  
1-3-1  
3-2-2  
3-3-1  
2-2-3  
1-2-3  
3-2-3  
2-2-1  
1-2-1  
3-2-1

**Probability**

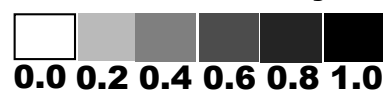

Novel Classes  
Known Classes

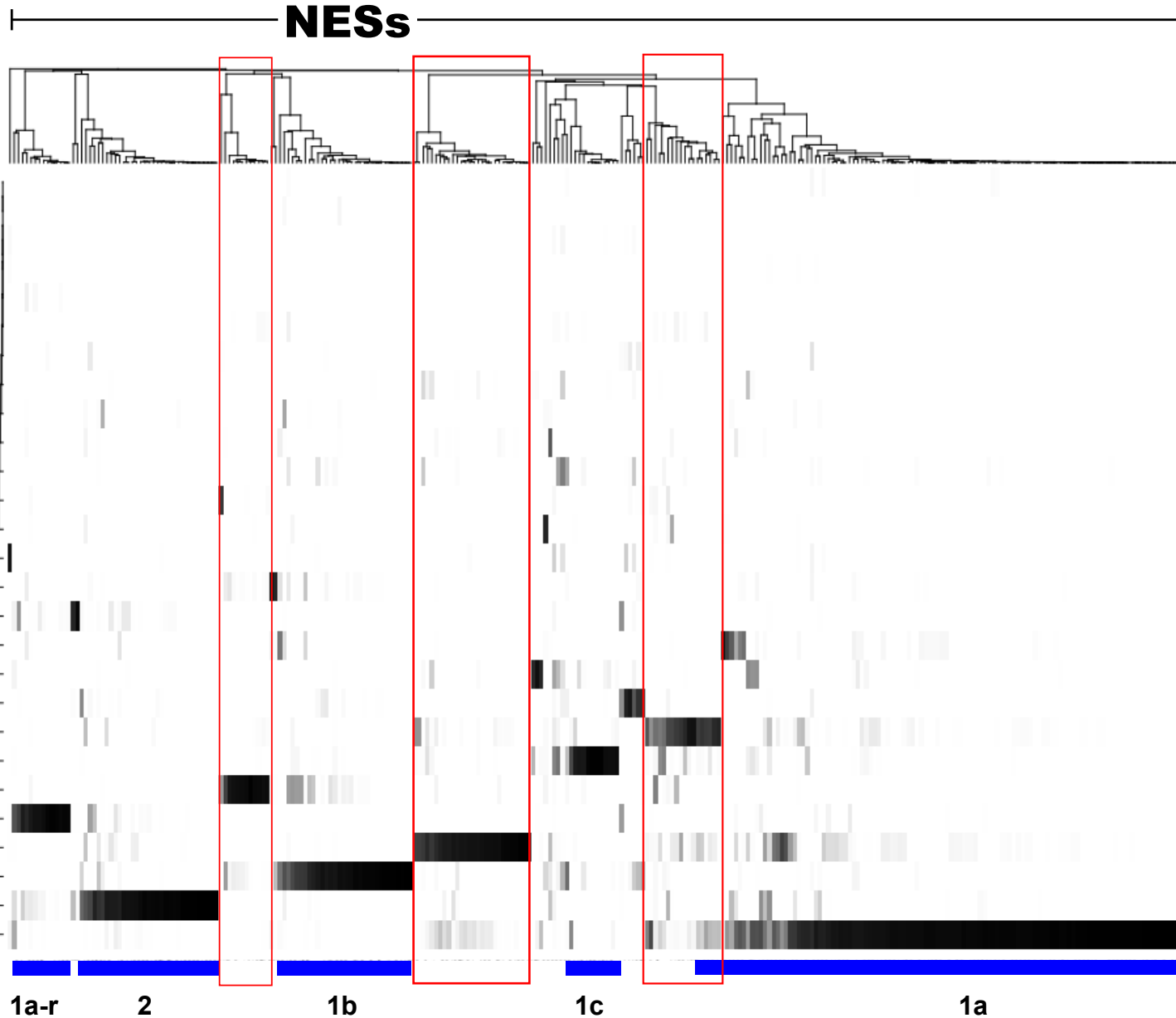

Supplement: Supplementary file 8 — Figure S6. This is the heat map generated from spacer configuration assignment probabilities of NESdb NESs using NoLogo with parameters estimated with the EM algorithm. (PDF 134 kb) [file 12859_2018_2076_MOESM8_ESM.pdf]

ROC curve NoLogo vs NoLogo NoNovel

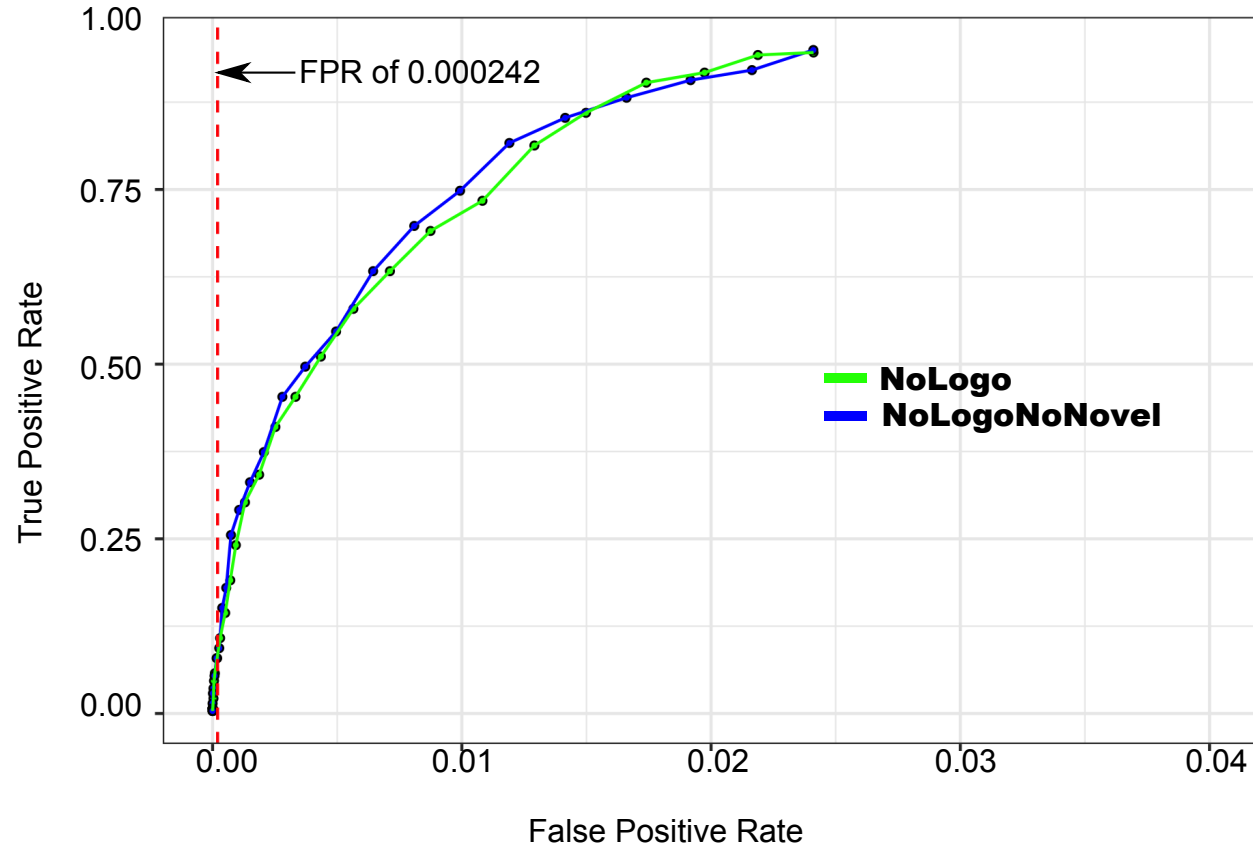

Supplement: Supplementary file 9 — Figure S7. NoLogo predictive performance not affected upon restriction to using only the 10 canonical classes as opposed to 27 possible spacer configuration categories. ROC curve analysis of NoLogo using all 27 possible spacer configurations (green line curve) vs 10 possible spacer configurations (purple line curve). (PDF 123 kb) [file 12859_2018_2076_MOESM9_ESM.pdf]

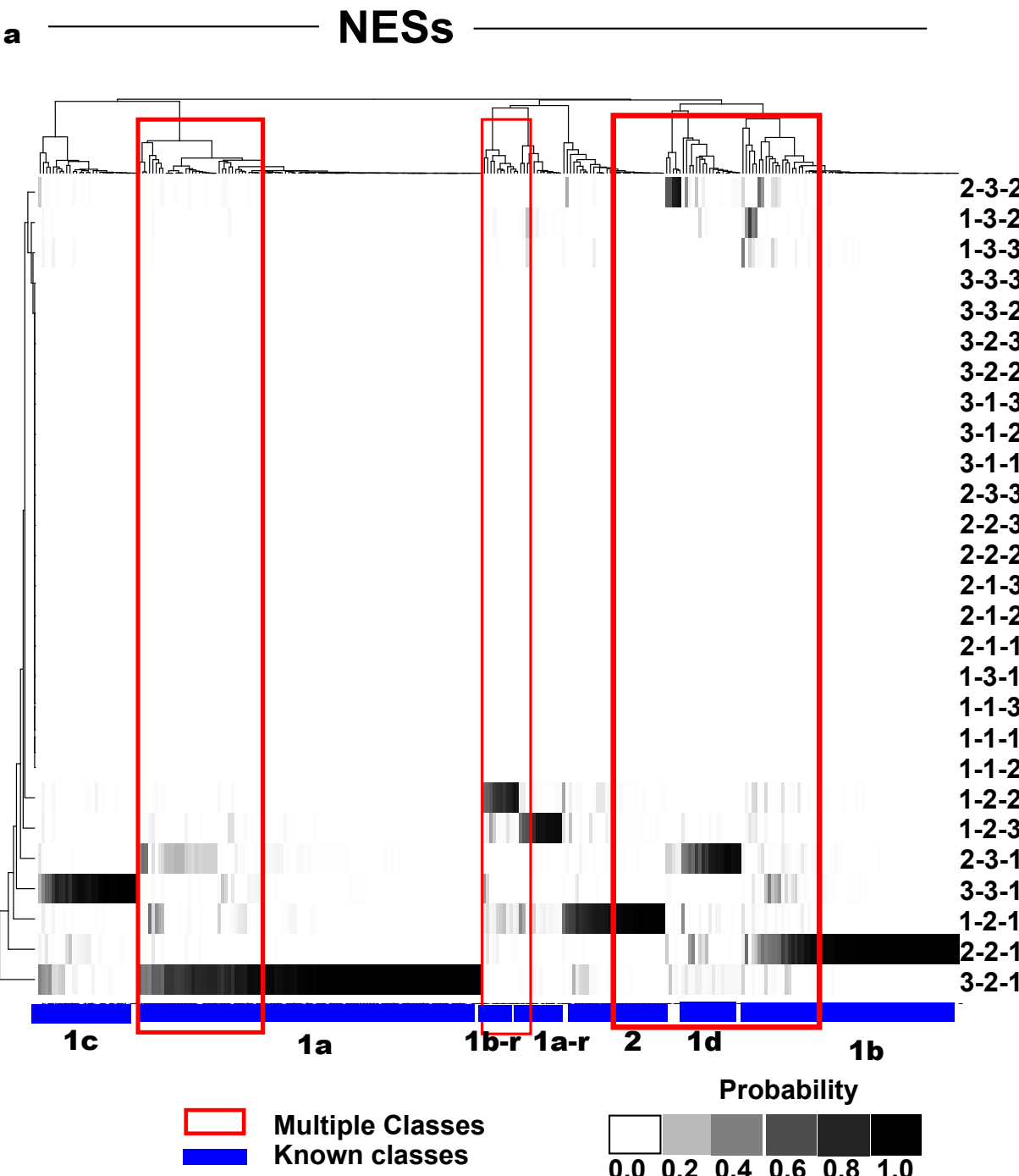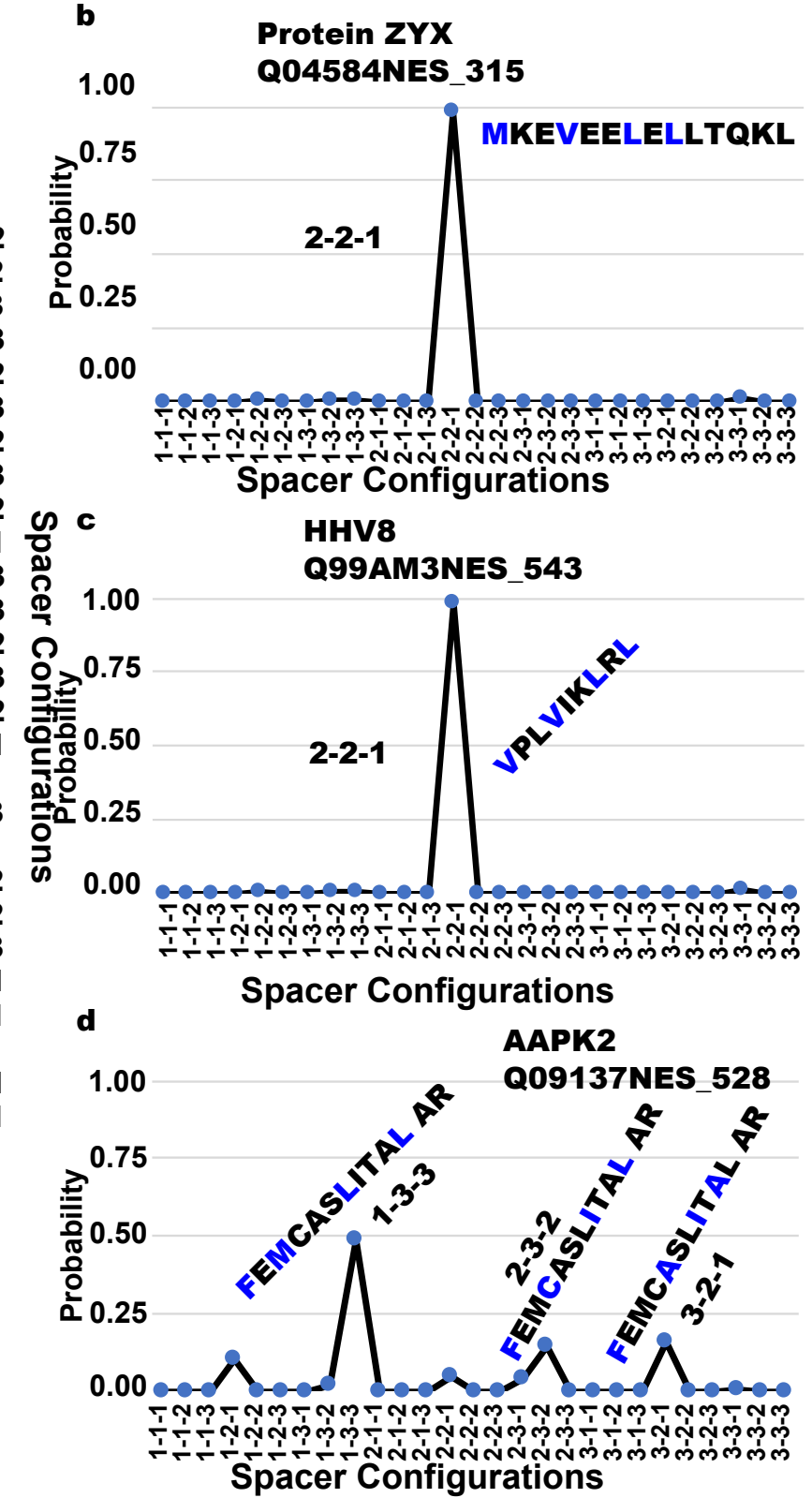

Supplement: Supplementary file 10 — Figure S8. NoLogo predictive performance not affected upon restriction to using only the 10 canonical classes as opposed to 27 possible spacer configuration categories. a) Posterior probabilities of NES configurations, using only the 10 canonical classes, are indicated in a heat map, where black corresponds to higher probabilities. Two hundred seventy-nine NESs have been clustered based on similarity, while configurations are ordered from least to most populated (top to bottom). Clusters corresponding to previously known classes of NESs are indicated as blue lines, while NESs exhibiting multiple classes are indicated as red boxes. b) The sample NES from Fig. 5 that is re-analyzed using settings that only allow 10 canonical spacer configurations. In Additional file 10: Figure S8b, this NES adopts 2–2-1 configuration whereas in Fig. 5b it exhibited 2–2-3 configuration. c) displays the posterior probability distribution of spacer configurations for an NES that exhibited up to 4 class membership with equal probabilities in Fig. 5c but now appears to be described by the 2–2-1 spacer configuration, solely, when NoLogo is restricted to just the 10 canonical spacer configuration classes. d) displays the posterior probability distribution of spacer configurations for an NES that exhibits up to 4 class membership, when NoLogo is restricted to just the 10 canonical spacer configuration classes. (PDF 938 kb) [file 12859_2018_2076_MOESM10_ESM.pdf]
