# Supplementary material for: NoLogo: a new statistical model highlights the diversity and suggests new classes of Crm1-dependent nuclear export signals
Source: BMC Bioinformatics. 2018 Feb 27;19:65. doi: 10.1186/s12859-018-2076-7 (PMC5828312; doi:10.1186/s12859-018-2076-7)
Supplement: Supplementary file 5 [file 12859_2018_2076_MOESM11_ESM.docx]

Table S2.
The frequency of each amino acid in the Saccharomyces cerevisiae proteome

A 0.05493370
C 0.01259810
D 0.05851865
E 0.06538412
F 0.04417271
G 0.04974184
H 0.02167693
I 0.06556848
K 0.07348676
L 0.09500875
M 0.02080609
N 0.06162645
